# Luteolin Is a Potential Immunomodulating Natural Compound against Pulpal Inflammation

**DOI:** 10.1155/2024/8864513

**Published:** 2024-01-25

**Authors:** Kentaro Kawakami, Takao Fukuda, Masaaki Toyoda, Yuki Nakao, Chikako Hayashi, Yukari Watanabe, Tsukasa Aoki, Takanori Shinjo, Misaki Iwashita, Akiko Yamashita, Miyu Shida, Terukazu Sanui, Takeshi Uchiumi, Fusanori Nishimura

**Affiliations:** ^1^Department of Periodontology, Division of Oral Rehabilitation, Faculty of Dental Science, Kyushu University, Fukuoka, Japan; ^2^Department of Clinical Chemistry and Laboratory Medicine, Graduate School of Medical Sciences, Kyushu University, Fukuoka, Japan

## Abstract

**Aim:**

The present study evaluated the therapeutic effects of luteolin in alleviating pulpitis of dental pulp- (DP-) derived microvesicles (MVs) via the inhibition of protein kinase R- (PKR-) mediated inflammation. *Methodology*. Proteomic analysis of immortalized human dental pulp (DP-1) cell-derived MVs was performed to identify PKR-associated molecules. The effect of luteolin on PKR phosphorylation in DP-1 cells and the expression of tumor necrosis factor-*α* (TNF-*α*) in THP-1 macrophage-like cells were validated. The effect of luteolin on cell proliferation was compared with that of chemical PKR inhibitors (C16 and 2-AP) and the unique commercially available sedative guaiacol-parachlorophenol. In the dog experimental pulpitis model, the pulps were treated with (1) saline, (2) guaiacol-parachlorophenol, and (3) luteolin. Sixteen teeth from four dogs were extracted, and the pulp tissues were analyzed using hematoxylin and eosin staining. Immunohistochemical staining was performed to analyze the expression of phosphorylated PKR (pPKR), myeloperoxidase (MPO), and CD68. Experimental endodontic-periodontal complex lesions were established in mouse molar through a silk ligature and simultaneous MV injection. MVs were prepared from DP-1 cells with or without pretreatment with 2-AP or luteolin. A three-dimensional microcomputed tomography analysis was performed on day 7 (*n* = 6). Periodontal bone resorption volumes were calculated for each group (nonligated–ligated), and the ratio of bone volume to tissue volume was measured.

**Results:**

Proteomic analysis identified an endogenous PKR activator, and a protein activator of interferon-induced PKR, also known as PACT, was included in MVs. Luteolin inhibited the expressions of pPKR in DP-1 cells and TNF-*α* in THP-1 cells with the lowest suppression of cell proliferation. In the dog model of experimental pulpitis, luteolin treatment suppressed the expression of pPKR-, MPO-, and CD68-positive cells in pulp tissues, whereas guaiacol-parachlorophenol treatment caused coagulative necrosis and disruption. In a mouse model of endodontic-periodontal complex lesions, luteolin treatment significantly decreased MV-induced alveolar bone resorption.

**Conclusion:**

Luteolin is an effective and safe compound that inhibits PKR activation in DP-derived MVs, enabling pulp preservation.

## 1. Introduction

Dental pulp tissues are highly susceptible to acute inflammation caused by various stimuli such as infection. Acute pulpitis results in rapid necrosis of the resident pulpal cells, followed by gangrene within a few days [1]. However, the underlying mechanisms leading to severe inflammation of the pulpal tissue are still not completely understood. Dental pulp is composed of soft connective tissue from dental pulp cells and vascular, lymphatic, and nervous elements [2]. Initial pulpal inflammatory response is mediated by innate effector immune cells. In particular, macrophages play critical role in the onset of pulpitis by producing tumor necrosis factor-*α* (TNF-*α*) [3]. Therefore, infiltration of macrophages is indispensable for inflammatory responses in pulpitis. In our *ex vivo* pulpitis model, not only primary dental pulp (DP) cells but also immortalized dental pulp (DP-1) cells exerted the prominent ability to induce TNF-*α* production in macrophages, which suggested that dental pulp cells secrete essential inducer molecules for pulpitis [4]. We denominated this unknown factor in the cell culture supernatant as dental pulp cell-derived powerful inducer of TNF-*α* (DPIT) and demonstrated that TNF-*α*-inducing activity of DPIT was stronger than that of lipopolysaccharide (LPS) [5]. Our previous study further identified that DPIT was derived from dental pulp cell-secreted microvesicles (MVs), and the activated protein kinase R (PKR) within the MVs was critical for DPIT activity [5]. Under pulpitis condition, dental pulp cells secrete MVs to transfer these activated PKRs into macrophages, resulting in the activation of inflammatory signaling pathways, such as nuclear factor- (NF-) *κ*B and c-Jun N-terminal kinase (JNK), and subsequent production of proinflammatory cytokines including TNF-*α* [6]. Although these results suggested that PKR inhibitors could be candidates for pulp sedative, existing chemical PKR inhibitors (C-16 and 2-AP) have not been approved for clinical use; meanwhile, the mechanism by which PKR is activated in DP-1 cells remains unclear. A thorough understanding of this mechanism is crucial to establish an effective pulp preservation strategy against inflammatory stimuli. Therefore, this study is aimed at (1) identifying the key molecules that activate PKR in DP-1 cells and (2) examining the efficacy of potential in vivo inhibitors using experimental animal models.

## 2. Materials and Methods

**Figure 1:**
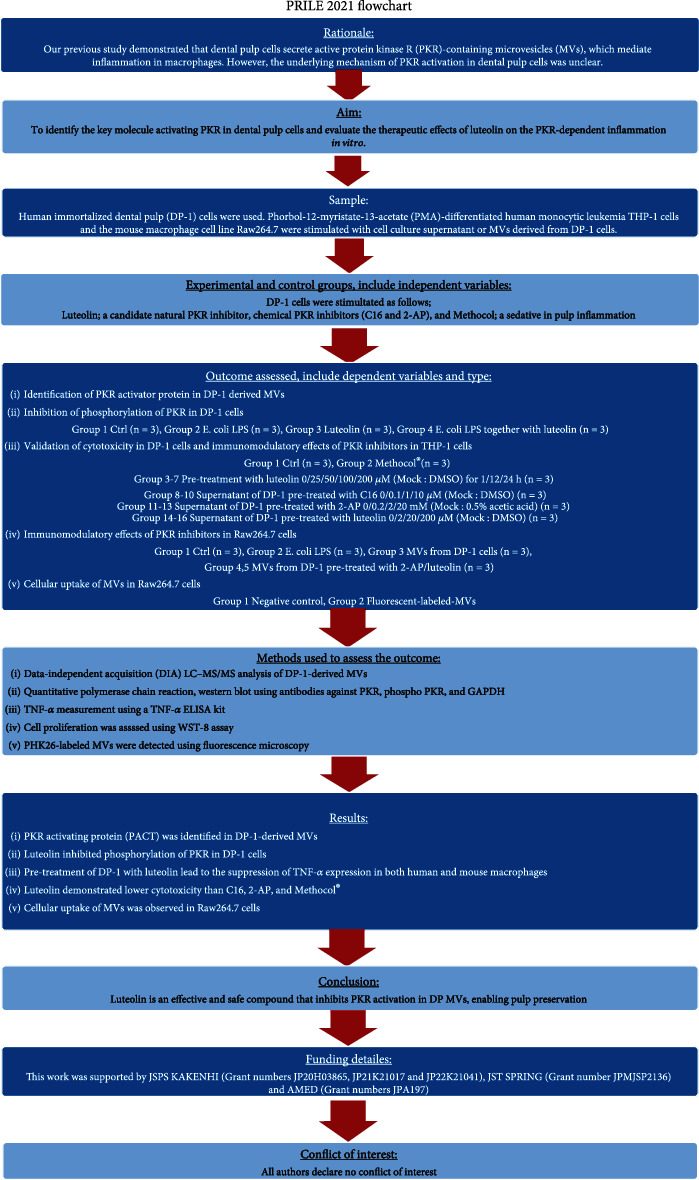
Flowchart according to preferred reporting items for laboratory studies in endodontology (PRILE) 2021 guidelines.

**Figure 2:**
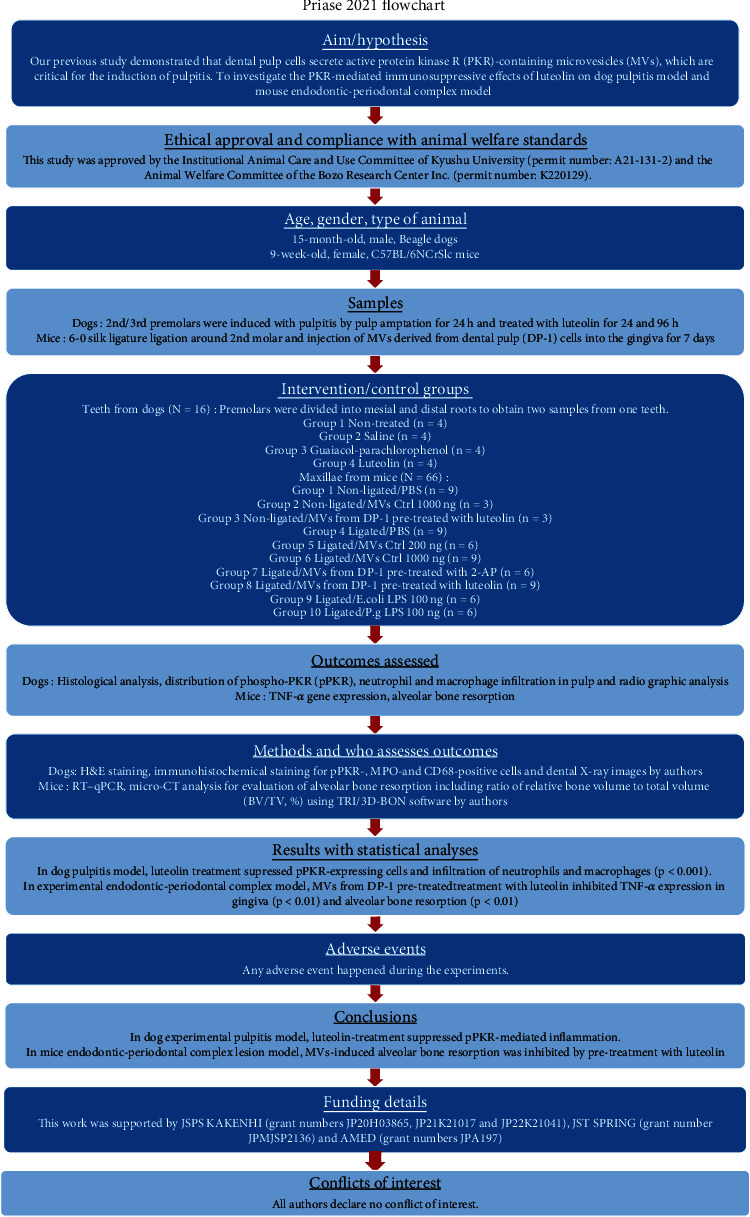
Flowchart according to preferred reporting items for animal studies in endodontology (PRIASE) 2021 guidelines.

The main study stages described in this manuscript (Figure 1) were written in accordance with the preferred reporting items for laboratory studies in endodontology (PRILE) 2021 guidelines [7] (Figure 2) and the preferred reporting items for animal studies in endodontology (PRIASE) 2021 guidelines [8].

### 2.1. Reagents

Luteolin was purchased from Cayman Chemical Co. (Ann Arbor, MI, USA). PKR inhibitors (2-aminopurine: 2-AP and C16) were purchased from Sigma–Aldrich (St. Louis, MO, USA). Phorbol-12-myristate-13-acetate (PMA) was purchased from Abcam (Cambridge, UK). Guaiacol-parachlorophenol (Methocol^®^), which is composed of 30% guaiacol and 70% parachlorophenol, was obtained from Neo Dental Chemical Products Co., Ltd. (Tokyo, Japan). Lipopolysaccharides (LPS) from *Escherichia coli* 055:B5 (*E. coli* LPS) and *Porphyromonas gingivalis* (*P. g* LPS) were purchased from Sigma–Aldrich.

### 2.2. Cell Culture

The establishment of immortalized human DP-1 cells has been described previously [9]. Human monocytic leukemia THP-1 cells (RCB1189) and the mouse macrophage cell line RAW264.7 were purchased from RIKEN Cell Bank (Tsukuba, Japan) and American Type Culture Collection (ATCC, Manassas, VA), respectively. DP-1 and RAW264.7 cells were cultured in Dulbecco's Modified Eagle Medium (Nacalai Tesque, Kyoto, Japan) supplemented with 10% fetal bovine serum (FBS) (Biowest, France), 100 U/ml penicillin, and 100 *μ*g/ml streptomycin. THP-1 cells were maintained in RPMI 1640 medium (Nacalai Tesque) containing 10% FBS, 2 mM GlutaMAX™ (Gibco, Thermo Fisher Scientific, Waltham, MA), and antibiotics.

THP-1 cells were seeded in 6-well plates at a density of 5 × 10^5^ cells/well and stimulated with 100 nM PMA (FUJIFILM Wako Pure Chemical, Osaka, Japan) for 24 h to obtain PMA-differentiated macrophages.

### 2.3. Isolation of MVs from DP-1 Cells

Isolation of MVs from DP-1 cells was performed according to our previous study [5]. Briefly, DP-1 cells were grown to subconfluency as described above. After the medium was aspirated, the cells were rinsed thrice with PBS and incubated for 24 h in fresh serum-free medium. The supernatant was collected, centrifuged at 2000 rpm for 20 min to remove debris and large apoptotic bodies, and filtered through an 800 nm filter (Sartorius Stedim Biotech, Göttingen, Germany). After the medium was further concentrated using 100 kDa Amicon Ultra-15 Centrifugal Filter Units (Merck Millipore, Billerica, MA, USA), the MVs were precipitated via centrifugation at 16,000 × g for 45 min, washed twice with phosphate-buffered saline (PBS; Nacalai Tesque), and dissolved in PBS containing 0.3 mM EDTA. The amount of protein in MVs was quantified using a BCA Protein Assay Kit (Takara Bio, Otsu, Japan).

### 2.4. Proteomic Analysis

MVs derived from DP-1 cells were subjected to data-independent acquisition (DIA) LC–MS/MS analysis by an assigned company (Hakarel, Osaka, Japan). A peptide false discovery rate (FDR) of <1% and a protein FDR of <1% were defined as thresholds for the identification of proteins [10]. The Mammalian Stress Granules Proteome (MSGP) database [11] was used for selecting stress granule component proteins.

### 2.5. WST-8 Cell Proliferation Assay

The WST-8 assay was performed as described previously [12]. DP-1 cells (5 × 10^3^ cells) in 100 *μ*l of growth medium were seeded into a 96-well culture plate. After 24 h, the medium was changed, and the cells were stimulated with PKR inhibitors (2-AP and C16), luteolin, and guaiacol-parachlorophenol. Thereafter, 10 *μ*l of WST-8 solution (Cell Count Reagent SF™: Nacalai Tesque) was added to each well (including the control wells) at 0 h, 24 h, 48 h, and 72 h. The cells were incubated for an additional 1 h at 37°C, following which the absorbance at 450 nm was measured, with a reference reading at 650 nm.

### 2.6. Enzyme-Linked Immunosorbent Assay (ELISA)

PMA-differentiated THP-1 cells were stimulated with the DP-1 supernatant with or without pretreatment with PKR inhibitors (2-AP and C16) or luteolin for 24 h. The expression levels of TNF-*α* were measured using the Human TNF-*α* Quantikine ELISA Kit (R&D Systems, Minneapolis, MN) according to the manufacturer's instructions.

### 2.7. Reverse Transcription–Quantitative Polymerase Chain Reaction (RT–qPCR) Analysis

RT–qPCR was performed as previously described [13]. Total RNA was isolated from THP-1 and RAW264.7 cells using ISOGENII (Nippon Gene, Tokyo, Japan). First-strand cDNA was synthesized using the PrimeScript RT Master Mix (Takara Bio, Otsu, Japan). RT–qPCR was performed on a StepOnePlus Real-Time System (Applied Biosystems, Carlsbad, CA, USA) using Luna® Universal qPCR Master Mix (New England Biolabs, Germany). The reaction conditions were as follows: initial denaturation at 95°C for 1 min, followed by 40 cycles of denaturation at 95°C for 15 s, and extension at 60°C for 30 s. Relative expression levels were calculated using the 2^−ΔΔCT^ method with the StepOne™ Software v2.3 (Applied Biosystems). Human GAPDH and mouse 18 s rRNA served as endogenous controls for THP-1 and RAW264.7 cells, respectively. Primer sequences used in this study are listed in Supplementary Table 1.

### 2.8. Western Blot Analysis

Western blotting was performed as described previously [14]. The cells were washed with PBS, lysed in Passive Lysis 5x buffer (Promega, Madison, WI, USA), and supplemented with a protease inhibitor cocktail (Nacalai Tesque). Protein samples were subjected to sodium dodecyl sulfate–polyacrylamide gel electrophoresis and transferred to polyvinylidene difluoride membranes (Millipore, Tokyo, Japan). The membranes were incubated with Blocking One-P (Nacalai Tesque) and probed with the following primary antibodies: anti-GAPDH (14C10) (1 : 1000, #2118; Cell Signaling Technology, Danvers, MA), anti-PKR (B-10) (1 : 1000, sc-6282; Santa Cruz Biotechnology, Dallas, TX), and anti-PKR (phospho T446) [E120] (1 : 200, ab32036; Abcam). Blotted membranes were detected using Chemi-Lumi One Super (Nacalai Tesque) and visualized using densitometry on an Amersham ImageQuant 800 platform (Cytiva, Tokyo, Japan). Immunoblots were quantified using Multi Gauge 3.1 software (FUJIFILM, Tokyo, Japan).

### 2.9. Dog Model of Experimental Pulpitis

Experiments in dogs were conducted at the BoZo Research Center (Shizuoka, Japan) after approval by the Animal Experiment Committee of the test facility and in accordance with the Institutional Animal Care and Use Committee (IACUC). Female beagle dogs (10.1–11.5 kg), aged 15 months, were purchased from Marshall BioResources (North Rose, NY) and subjected to two weeks of quarantine and acclimation. The dogs were premedicated with intramuscular atropine sulfate injection (0.5 mg/1 ml/body) (Mitsubishi Tanabe Pharma, Osaka, Japan) and anesthetized with intravenous administration of 1% propofol (7.5 mg/kg) (Nichi-Iko Pharmaceutical Co., Ltd., Toyama, Japan). Dogs undergoing endotracheal intubation during the procedure were mechanically ventilated and anesthetized with isoflurane (Isoflurane Inhalation Solution, Pfizer, Tokyo, Japan). Experimental pulpitis was induced in the dogs according to a previously established model [15]. After administration of local anesthesia using 2% lidocaine with 1 : 80,000 epinephrine (0.9 ml/teeth) (Xylocaine Cartridge for Dental Use, Dentsply Sirona, Tokyo, Japan), the crowns of the upper and lower premolars were extracted with a diamond point burr (Shofu, Tokyo, Japan). Subsequently, the pulp tissues were amputated with a no. 1/2 round burr (Shofu). Amputated pulp tissues were exposed to oral bacteria for 24 h to induce pulpitis. After pulpotomy, the mesial and distal root canal orifices were treated with saline-, luteolin-, or guaiacol-parachlorophenol-soaked sterile cotton balls. The cavities were sealed with a light-cured glass ionomer cement (Ionosit-Baseliner; DMG America, Ridgefield Park, NJ, USA) and a dual-cure resin composite (ESTE CORE, Tokuyama Dental, Tokyo, Japan) after treatment with a bonding agent (G-Premio BOND, GC, Tokyo, Japan). After sealing the cavities for 24 or 96 h, the dogs were euthanized, and the test teeth were extracted.

### 2.10. Dental X-Ray Images

Before pulp amputation, capping, and tooth extraction, X-ray images of the teeth were obtained using a portable X-ray system (X-Shot; YOSHIDA Dental Trade Distribution Co., Ltd., Tokyo, Japan) and an ISO Speed D X-ray dental film (DIC-100; Hanshin Technical Laboratory, Nishinomiya, Japan) with photograph indicators (Cone Indicator-S; Hanshin Technical Laboratory).

### 2.11. Histology and Immunohistochemistry

Sixteen teeth from four dogs were extracted. Premolars were divided into mesial and distal roots to obtain two samples from one tooth. The extracted tooth sample was fixed in 4% paraformaldehyde (PFA) at 4°C for 24 h, decalcified with Kalkitox™ at 4°C for 48 h, neutralized in 5% sodium sulfate solution for 24 h (all purchased from FUJIFILM Wako Pure Chemical), and embedded in paraffin. Standard hematoxylin and eosin (H&E) staining was performed as previously described [16]. For immunohistochemical staining, the specimens were probed with specific primary antibodies against myeloperoxidase (MPO) (1 : 1000, ab208670; Abcam), CD68 (1 : 150, E3O7V; Cell Signaling Technology), and pPKR (phospho T446) (1 : 200, ab32036; Abcam) and counterstained with Mayer's hematoxylin solution (FUJIFILM Wako Pure Chemical).

### 2.12. Mouse Model of Endodontic-Periodontal

C57BL/6NCrSlc mice (female, 8-week-old) were purchased from Japan SLC (Hamamatsu, Japan) and used following the guidelines of an institutionally approved animal research protocol (protocol #A21-131-2; Kyushu University). After one week of quarantine and acclimation, ligature-induced periodontal mouse model was established as previously described [17]. Briefly, the mice were randomly classified into the following four groups with different treatments (*n* = 6): (1) placebo (PBS), (2) DP-1-derived MVs, (3) luteolin-treated DP-1-derived MVs, and (4) LPS from *E. coli* or *P. gingivalis*. To induce periodontal bone loss in the mice, a 6-0 silk ligature (Akiyama Medical MFC Co., Tokyo, Japan) was tied around the right maxillary second molar. After ligation, placebo (PBS) or MVs were injected into the palatal gingiva of the maxillary second molar using a 33-gauge needle Hamilton syringe (Hamilton Company, NV).

### 2.13. Immunofluorescence Analysis

The MVs were fluorescently labeled using PKH26 (Sigma–Aldrich) as previously described [5]. The red fluorescence-labeled MVs were incubated with RAW264.7 cells at a final concentration of 10 *μ*g/ml and incubated for 1 h. Following incubation, the cells were washed with PBS and fixed with 4% PFA. To detect locally injected MVs in mice, maxillae were fixed in 4% PFA for 24 h, demineralized using Osteosoft solution (Merck, Darmstadt, Germany) for 72 h, and embedded in paraffin. The slides were covered with ProLong Diamond antifade mountant containing DAPI (Thermo Fisher Scientific, Waltham, MA), and the images were captured using a fluorescence microscope (Keyence Co., Osaka, Japan).

### 2.14. Microcomputed Tomography (Micro-CT) Scanning

The maxillae of mice were scanned using a micro-CT imaging system (ScanXmate; Comscan, Kanagawa, Japan), and three-dimensional (3D) images were reconstructed using TRI/3D-BON software (Ratoc System Engineering, Tokyo, Japan). Periodontal bone resorption analysis was performed using a split-mouth experimental design: one side of the maxilla was ligated and locally injected with the MVs or LPS, whereas the other side without ligation served as the control. Alveolar bone resorption volumes, including the ratio of bone volume to total volume (BV/TV%), were evaluated for each group (nonligated–ligated) as previously described [17].

### 2.15. Statistical Analysis

Data were presented as mean ± standard deviation. Data analyses were performed using the GraphPad Prism 9 software (GraphPad Software Inc., La Jolla, CA, USA). One-way or two-way analysis of variance was performed, followed by correction for multiple comparisons using Tukey's post hoc test to compare three or more groups for analyzing statistical significance. Other statistical comparisons of data between the two groups were performed using the Student's *t*-test. Statistical significance was set at *p* < 0.05.

## 3. Results

### 3.1. Identification of Endogenous PKR Activator in Dental Pulp Cell-Derived MVs by Proteomic Analysis

**Figure 3:**
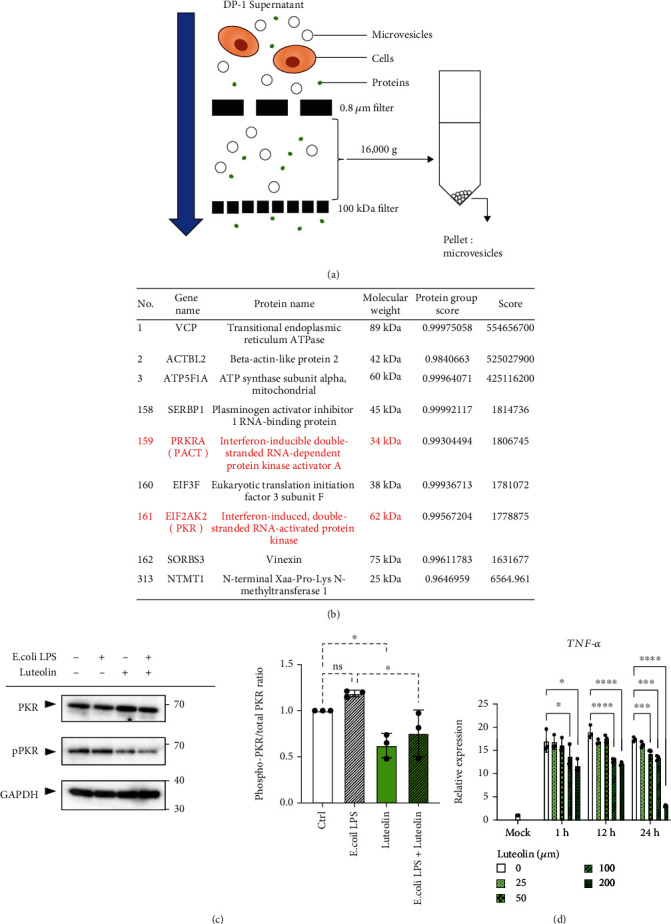
Identification of an endogenous PKR activator, a protein activator of interferon-induced PKR (PACT), using proteomic analysis and the inhibition of PKR phosphorylation by luteolin. (a) Schematic procedure for the isolation of microvesicles (MVs) from cell culture supernatant of dental pulp (DP-1) cells. (b) Representative stress granule component proteins in DP-1-derived MVs. Proteins from DP-1-derived MVs were identified using data-independent acquisition (DIA) proteomic analysis, subsequently followed by selection of stress granule component proteins using the Mammalian Stress Granules Proteome (MSGP) database (https://msgp.pt/). (c) Luteolin inhibits lipopolysaccharide- (LPS-) induced phosphorylation of protein kinase R (PKR) in DP-1 cells. DP-1 cells with or without luteolin (100 *μ*M) pretreatment for 24 h were stimulated with *E. coli* LPS (100 ng/ml) for 3 h. GAPDH was used as the internal control (left). Relative phosphorylation of PKR was measured by quantifying the density of phospho-PKR to the densitometry of total PKR (*n* = 3) (right). (d) Luteolin inhibits DP-1 supernatant-induced expression of TNF-*α* mRNA in PMA-differentiated THP-1 (dTHP-1) cells. After pretreatment of DP-1 with the indicated concentration of luteolin for 24 h, dTHP-1 cells were stimulated with the supernatants derived from DP-1 for 3 h (*n* = 3). ^∗^*p* < 0.05, ^∗∗∗^*p* < 0.001. ^∗∗∗∗^*p* < 0.0001. Error bars represent means ± SD. Data were analyzed using independent unpaired two-tailed Student's *t*-tests.

To explore PKR-activating molecules in dental pulp cell-derived MVs (DP-MVs), we performed proteomic analysis (Figures 3(a) and 3(b)). Among the 4189 identified proteins in DP-MVs, we found 313 stress granule component proteins using MSGP, the database for the protein components of mammalian stress granules (https://msgp.pt/) (Supplementary Table 2). Nine representative stress granule component proteins were summarized in Figure 3(b). Specifically, we focused on the PKR-activating protein (PACT), which is the known endogenous PKR activator [18]. To examine the role of PACT in PKR activation, luteolin was used to block the interactions between these proteins. In DP-1 cells, luteolin significantly inhibited phosphorylation of PKR under LPS stimulation (Figure 3(c)). In PMA-differentiated THP-1 cells, stimulation with luteolin-free conditioned medium from DP-1 cells enhanced the expression of TNF-*α* mRNA. However, pretreatment of DP-1 with luteolin dose- and time-dependently decreased the expression of TNF-*α* mRNA (Figure 3(d)).

### 3.2. Comparison of Immune-Modulating Effect of Luteolin with Known Inhibitors

**Figure 4:**
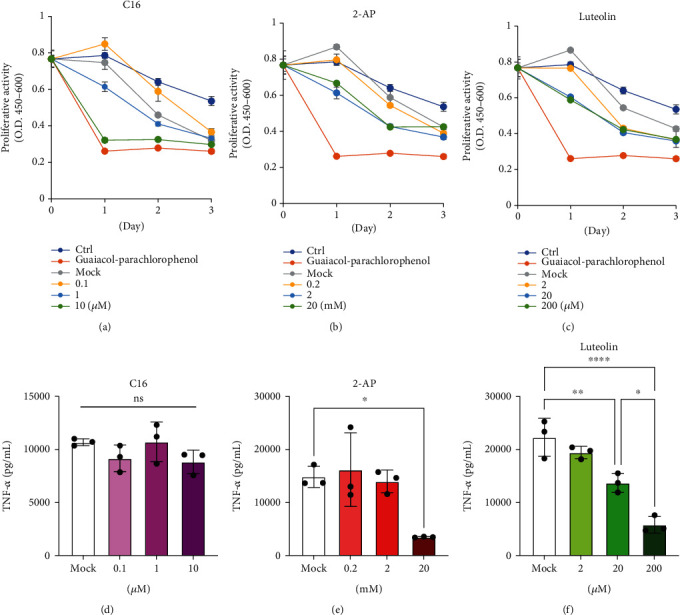
Luteolin induces superior inhibition of TNF-*α* expression with less suppression of cell viability compared with C-16 and 2-AP. (a–c) The effect of protein kinase R (PKR) inhibitors (C16 and 2-AP) and luteolin on cell proliferation of DP-1 cells was validated using WST-8 assay. Control indicates both: no stimulation (Ctrl) and treatment with guaiacol-parachlorophenol (0.1 vol %). Dimethyl sulfoxide (DMSO) for C16 and luteolin or 0.5% acetic acids for 2-AP were used as mock solvent control (Mock). Proliferation activity was expressed as the absorbance at 450 nm minus the absorbance at 650 nm. (d–f) Comparison of immune-modulating effects between PKR inhibitors (C16 and 2-AP) and luteolin. After pretreatment with C16, 2-AP, and luteolin for 24 h, dental pulp (DP-1)-supernatant-induced TNF-*α* production in dTHP-1 cells was quantified using enzyme-linked immune sorbent assay (ELISA) (*n* = 3). ^∗^*p* < 0.05, ^∗∗^*p* < 0.01. ^∗∗∗∗^*p* < 0.0001. Error bars represent means ± SD. Data were analyzed using independent unpaired two-tailed Student's *t*-tests.

We investigated the potential application of luteolin in pulpitis *in vitro*. The effects of luteolin on cell proliferation in DP-1 cells and anti-inflammatory effects in PMA-differentiated THP-1 cells were compared with those of existing chemical PKR inhibitors (C-16 and 2-AP). Guaiacol-parachlorophenol, the unique phenolic dental pulp sedation agent, was used as a control. Cell viability assay revealed the applicable concentrations for C16 (<1 *μ*M), 2-AP (<20 mM), and luteolin (<200 *μ*M), whereas guaiacol-parachlorophenol revealed far stronger inhibition of cell proliferation at day 1 (Figures 4(a)–4(c)). After pretreatment with these inhibitors for 24 h, DP-1-supernatant-induced TNF-*α* productions in PMA-differentiated THP-1 cells were significantly inhibited by 2-AP (20 mM) and luteolin (20 and 200 *μ*M), while C16 had no effect (Figures 4(d)–4(f)). Furthermore, the TNF-*α* inhibition effect of 20 mM 2-AP was almost equivalent to 200 *μ*M luteolin. These results indicate that luteolin exerts immune-modulating effect with lower concentration and inhibition of cell viability than C16, 2-AP, and guaiacol-parachlorophenol.

### 3.3. In Vivo Effects of Luteolin on the Integrity of Pulp Tissue in Dog Experimental Pulpitis Model

**Figure 5:**
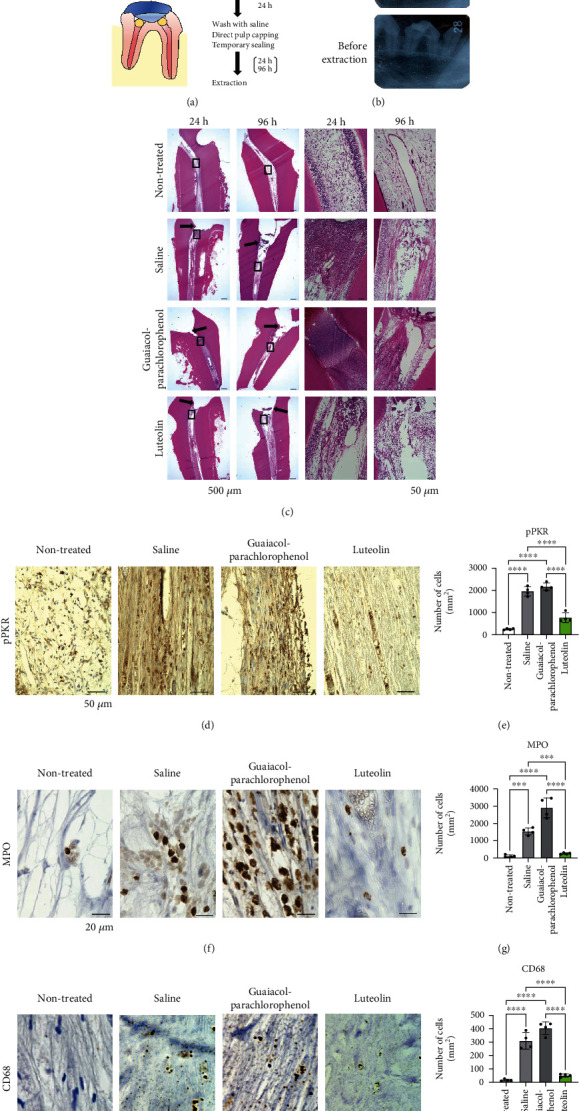
Luteolin suppresses the onset of pulpitis in dog model of experimental pulpitis. (a) Schematic illustration representing dog experimental pulpitis model. After pulpotomy of the upper and lower premolars for 24 h, the root canal orifices were washed with saline and treated with luteolin- or guaiacol-parachlorophenol-soaked sterile cotton ball. After sealing the cavities for 24 or 96 h, the test teeth were extracted. (b) Dental X-ray images were captured before pulp amputation, pulp capping, and tooth extraction. (c) Hematoxylin and eosin (H&E) staining of pulp tissues was performed at 24 and 96 h after pulp capping. The arrows indicate the amputated site. Black boxes indicate magnified views. Scale bar = 500 *μ*m and 50 *μ*m for low (left) and high magnification, respectively. (d) Immunohistochemical staining of phosphorylated-PKR (pPKR) in pulp tissues. (e) The number of pPKR-positive cells in pulp tissue. Cell counts are indicated as positive cells per square millimeter. (f) Immunohistochemical staining of myeloperoxidase (MPO) in pulp tissues. (g) The number of MPO-positive cells in pulp tissue. Cell counts are indicated as positive cells per square millimeter. (h) Immunohistochemical staining of CD68 in pulp tissues. (i) The number of CD68-positive cells in pulp tissue. Cell counts are indicated as positive cells per square millimeter. Error bars represent the mean ± SD, *n* = 4. ns: not significant; ^∗∗∗^*p* < 0.001, ^∗∗∗∗^*p* < 0.0001. The statistical significance of differences between groups was determined using one-way ANOVA, followed by correction for multiple comparisons using Tukey's post hoc test.

To validate the therapeutic effects of luteolin in vivo, we established a canine pulpitis model (Figure 5(a)). The amputated pulps were treated with luteolin or guaiacol-parachlorophenol, and saline was used as the control. Dental radiographs confirmed successful pulp exposure at the root canal orifices, and the cavity was sealed until tooth extraction (Figure 5(b)). Histological analysis of pulp tissues revealed significant neutrophil infiltration at the amputation site. In the guaiacol-parachlorophenol-treated group, pulp tissues were coagulative necrosis at 24 h and exhibited enhanced disruption at 96 h. In contrast, the luteolin-treated group maintained their morphology for 96 h without disruption or necrosis (Figure 5(c)). Since PKR-mediated inflammation is closely associated with phosphorylated-PKR (pPKR), and MPO is an indicator of inflammation, we further evaluated the expression of pPKR, MPO, and CD68 by immunohistochemical staining (Figures 5(d)–5(i)). High numbers of pPKR-, MPO-, and CD68-positive cells were observed in both the control (saline) and guaiacol-parachlorophenol. However, luteolin treatment significantly decreased the number of pPKR-positive cells by approximately one-third, and the number of MPO- and CD68-positive cells was almost the same as those in the control. To support this notion, luteolin suppressed LPS-induced mRNA expressions of interleukin-6 (IL-6), monocyte chemoattractant protein-1 (MCP-1), and interleukin-8 (IL-8) in DP-1 cells (Supplementary Figure 1). Collectively, these results suggest that luteolin suppresses the onset of pulpitis by inhibiting pPKR-mediated pulpal inflammation.

### 3.4. In Vivo Effects of Luteolin on Alveolar Bone Loss in Experimental Endodontic-Periodontal Complex Lesion in Mice

**Figure 6:**
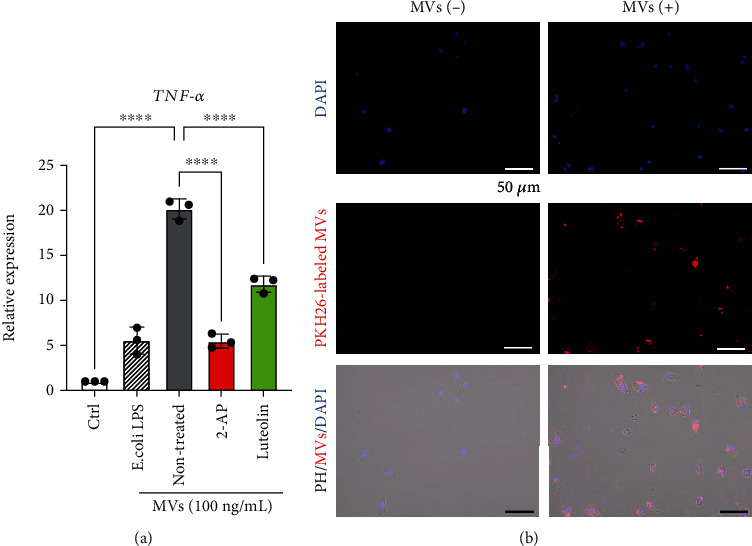
Luteolin downregulates DP-1-MV-induced TNF-*α* expression in murine macrophage RAW264.7 cells. (a) Luteolin inhibits microvesicles (MVs) from dental pulp cell- (DP-1-) induced expression of TNF-*α* mRNA in RAW264.7 cells. After pretreatment of DP-1 with 2-AP (10 mM) or luteolin (100 *μ*M) for 24 h, RAW264.7 cells were stimulated with the MVs derived from DP-1 or *E. coli* LPS (100 ng/ml) for 3 h (*n* = 3). ^∗∗^*p* < 0.01, ^∗∗∗^*p* < 0.001, ^∗∗∗∗^*p* < 0.0001. Error bars represent means ± SD. Data were analyzed using independent unpaired two-tailed Student's *t*-tests. (b) Cellular uptake of DP-1-derived MVs by RAW264.7 cells was observed using fluorescent microscopy. PKH26-labeled MVs (red) were incubated with murine macrophages for 1 h. Cell nuclei were stained with DAPI (blue). Bottom panel shows merged images, including phage contrast (PH) images. Scale bar = 50 *μ*m.

**Figure 7:**
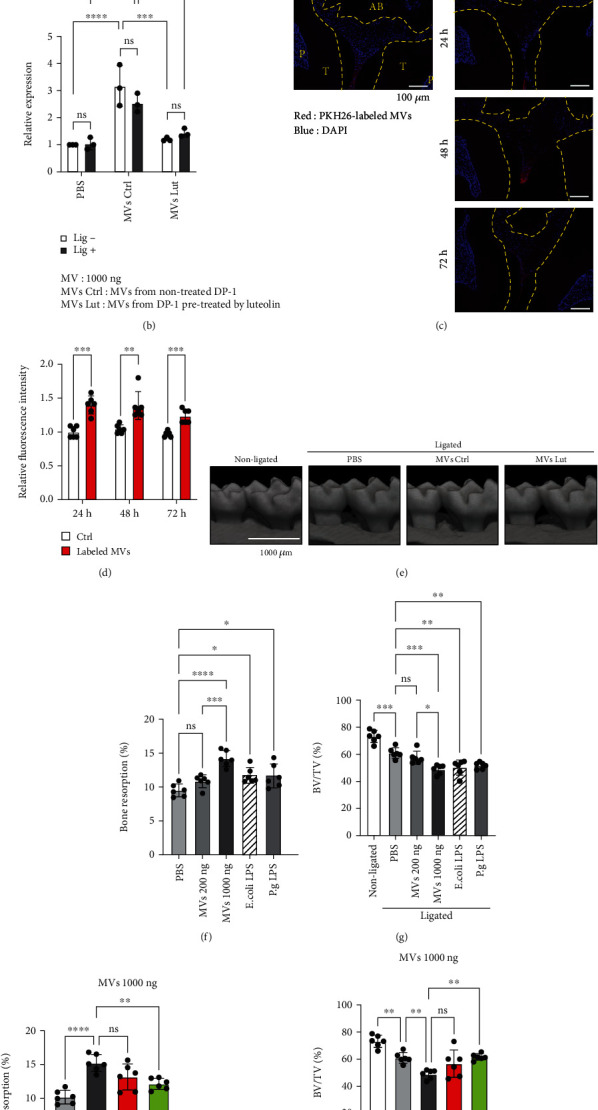
Luteolin inhibits alveolar bone loss in a mouse model of experimental endodontic-periodontal complex lesions. (a) Schematic illustration of the experimental endodontic-periodontal complex lesion model in C57BL/6 mice. A 6-0 silk ligature was tied around the maxillary second molar, and microvesicles (MVs) derived from dental pulp (DP-1) cells were simultaneously injected as illustrated. (b) On day 1, the mRNA expression of TNF-*α* in gingival tissue was analyzed. Mice were injected with 1000 ng of DP-1-derived MVs with (MV Lut) or without (MV Ctrl) luteolin pretreatment. (c) Time course retention of injected MVs in the mouse periodontal tissue was monitored using fluorescent microscopy. MVs (1000 ng) were labeled with PKH26 (red) before administration. Nuclei were stained with DAPI (blue). AB: alveolar bone; T: tooth; P: pulp. (d) Intensity of PKH-26-labeled fluorescent MVs at 24, 48, and 72 h after injection was measured. (e) Three-dimensional microcomputed tomography (micro-CT) images of the maxillae of each treatment group on day 7 after ligature placement. Scale bar = 1,000 *μ*m. (f–i) Periodontal bone resorption analysis was performed using a split-mouth experimental design: one side of the maxilla was ligated and locally injected with the indicated amount of MVs or 100 ng of LPS from *E. coli* and *P. g* (f, g), whereas the other side without ligation served as the control. (f, h) Relative alveolar bone resorption volume was calculated for each group (nonligated–ligated). (g, i) The ratio of bone volume to tissue volume (BV/TV). Error bars represent the mean ± SD, *n* = 3 (b), 6 (d, f–i). ns: not significant, ^∗^*p* < 0.05, ^∗∗^*p* < 0.01, ^∗∗∗^*p* < 0.001, ^∗∗∗∗^*p* < 0.0001. The statistical significance of differences between groups was determined using one-way ANOVA, followed by correction for multiple comparisons using Tukey's post hoc test.

To gain further insight into dental pulp cell (DP-1)-MV-mediated inflammation in periodontal tissue, we postulated that MVs could exacerbate alveolar bone loss in a mouse periodontal model. To validate whether human DP-MVs also cause inflammation in mice, RAW264.7, cells were stimulated with DP-1-MVs with or without pretreatment with PKR-inhibitors (2-AP and luteolin). In RAW264.7 cells, DP-1-MV stimulation significantly enhanced the expression of TNF-*α* mRNA (20.2-fold), which was greater than LPS stimulation (5.53-fold), while it was reduced by pretreatment with 2-AP and luteolin (Figure 6(a)). We next labeled DP-1-MVs with a fluorescent dye and observed their cellular uptake in RAW264.7 cells (Figure 6(b)). These results led us to hypothesize that the injection of DP-MVs into the gingiva in a ligature-induced periodontal model could establish an experimental endodontic-periodontal complex model in mice (Figure 7(a)). In mouse gingiva, injection of DP-MVs increased the expression of TNF-*α* mRNA, and it was significantly decreased by the pretreatment of MVs with luteolin (Figure 7(b)), as observed *in vitro* (Figure 6(a)). Fluorescent imaging revealed that the locally injected MVs were retained in the gingival tissue for 72 h (Figures 7(c) and 7(d)). To investigate the inhibitory and therapeutic potential of luteolin against alveolar bone loss in this model, a micro-CT analysis was performed (Figures 7(e)–7(i)). Injection of 1000 ng of MVs caused higher bone resorption compared to 200 ng of MVs and 100 ng LPS from *E. coli* and *P. g* (Figures 7(f) and 7(g)). However, MVs pretreated with luteolin significantly attenuated the bone resorption activity (Figures 7(h) and 7(i)). Collectively, these results suggest that the luteolin-mediated inactivation of PKR in MVs is responsible for the inhibition of bone loss in the endodontic-periodontal complex model.

## 4. Discussion

In our previous study [5], we could not identify PKR-activating molecules by proteomic approach and thought this might be due to the fact that we previously used entire cell supernatant for proteomic analysis. Therefore, in this study, we isolated MVs from the supernatants of dental pulp cells and subjected these concentrated MVs to proteomic analyses. We, for the first time, identified PKR-activating protein (PACT) as a known endogenous PKR activator [19] from dental pulp cells. PACT is an endogenous protein directly binding to and activating PKR, resulting in the activation of subsequent proinflammatory signals including NF-*κ*B and JNK [20]. Therefore, we hypothesized that this molecule is a key upstream protein that induces severe pulpal inflammation.

Next, we attempted to identify potential PKR inhibitors and found an important study reporting that luteolin, a polyphenolic flavone, was a natural inhibitor of PKR that has been reported to directly inhibit the binding of PACT to PKR [21–23]. Thus, we decided to examine the effects of luteolin on its immunomodulatory activity by comparing the effects of known chemical PKR inhibitors, such as C16 [24] and AP-2 [25], both of which are not recommended for in vivo use. Interestingly, the effects of luteolin were comparable to those of these inhibitors and even higher than those of C16 in terms of the suppression of TNF-*α* gene expression in macrophage-like PMA-stimulated THP-1 cells. Furthermore, considering that 200 *μ*M of luteolin significantly inhibited TNF-*α* production than that of 20 *μ*M of luteolin under the same cell proliferative activities (Figure 4), suggesting that luteolin exerted superior immunomodulatory effect independent of cell viability in DP-1 cells. Luteolin is a natural polyphenolic flavone present in some vegetables or plants and is already being used as an oral supplement [26]. Meanwhile, our previous study revealed that TNF-*α* stimulates the productions of IL-6 and MCP-1 in dental pulp cells [4]. Besides the proinflammatory properties of TNF-*α* and IL-6, MCP-1 contribute to macrophage infiltration [27]. Thus, we hypothesized that luteolin would be an ideal natural compound to confirm its immunomodulatory effects in pulpitis.

Next, we examined the immunosuppressive effects of luteolin in vivo. We used two experimental models, a dog pulpitis model and a mouse endodontic-periodontal complex model. We observed the effects of luteolin on the prevention of pulpal inflammation in dog molars. As control, the only commercially available sedative disinfectant, Methocol®, which contained 30% guaiacol and 70% parachlorophenol was used. These results indicated that luteolin-treated dog pulp tissue cells did not undergo cell death when the pulp tissue was directly exposed to artificial drilling. Histological observations revealed that the appearance of the luteolin-treated pulp tissue was almost the same as that of the untreated tissues, even though massive bacterial invasion was observed after drilling. The control guaiacol-parachlorophenol-treated pulp tissue underwent complete loss, possibly because of the toxic effects of parachlorophenol. The positive control (pulp exposed to saline) also showed signs of severe inflammation. Thus, it appeared that luteolin could protect the pulp tissue from necrosis after pulp exposure. Luteolin was reported to exert superior antimicrobial activity against oral *Streptococci* and *P. gingivalis* [28], antibiofilm effects [29], and the neuroprotective effects [30]. Taken together, these effects of luteolin could contribute to the inhibition of pulpitis in our “mild” pulpitis model [15], which was caused by 24 h exposure of amputated pulp, followed by sealing of the cavities.

Next, we examined whether dental pulp cell-derived MVs could slow the breakdown of mouse periodontal tissue in an experimental endodontic-periodontal complex model. Since it was unclear that mouse pulp cells also produce stress granule-containing MVs outside the cells, hence, the pathological mechanisms share the common feature as human, and it was technically very difficult to induce pulp exposure model in mouse molars; we directly injected MVs derived from human dental pulp cells into mouse gingiva in the experimental mice which were induced with ligature-induced experimental periodontitis. Since combined endodontic-periodontal lesions can be caused by primary periodontal disease with secondary endodontic involvements (so-called ascending pulpitis) [31], we thought this model partially mimic endodontic-periodontal complex as this was the combination of both MV-induced and ligature-induced inflammation, and we evaluated the degree of alveolar bone resorption. These results indicated that luteolin suppressed alveolar bone loss in mouse periodontal tissues.

## 5. Conclusion

**Figure 8:**
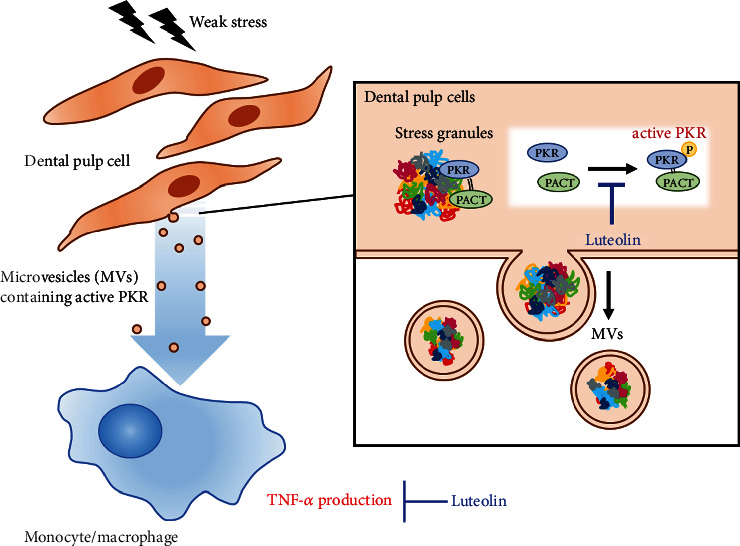
Luteolin induces its therapeutic effect on pulpitis of DP-1-derived MVs by inhibiting the activation of PKR. In dental pulp cells, weak stress signals induce protein kinase R (PKR) phosphorylation, resulting in the release of active PKR-containing microvesicles (MVs), which function as an extremely powerful TNF-*α* inducer in monocytes and macrophages (left). PKR-activating protein (PACT), an endogenous PKR activator, enhances PKR phosphorylation in stress granule complexes. Luteolin antagonizes the interaction between PKR and PACT to inhibit PKR activation, which could be a potential strategy for suppressing the onset of pulpitis (right).

In summary, we first identified that DP-derived MVs contained PACT, a known endogenous PKR activator by proteomic analysis. Based on the possible mechanism that luteolin antagonizes the interaction between PKR and PACT to inhibit PKR activation, which is critical for MV-induced TNF-*α* induction in macrophages and subsequent onset of pulpitis, we validated the therapeutic effect of luteolin on pulpal inflammation. In the dog experimental pulpitis model, we demonstrated for the first time that luteolin inhibits the activation of PKR in dental pulp MVs to exert superior immunomodulatory effects. Our findings, together with the previously reported mechanisms [5], are summarized in Figure 8. Based on our current investigation, we believe that luteolin is an effective and safe compound for pulp preservation and is, therefore, suitable for in vivo use.

## Data Availability

The data used to support the findings of this study are available from the corresponding author (Takao Fukuda) on request.

## Supplementary Materials

Supplementary 1 Supplementary Table 1: primer sequence used for quantitative RT-PCR. Supplementary Table 2: stress granule-related proteins in MVs from DP-1 detected by proteomic analysis.

Supplementary 2 Supplementary Figure 1: luteolin downregulates LPS-induced mRNA expressions of MCP-1, IL-6, and IL-8 in dental pulp cells. DP-1 cells were stimulated with *E. coli* LPS (10 ng/ml) for 3 h with or without pretreatment with luteolin (200 *μ*M) for 24 h (*n* = 3). ^∗∗^*p* < 0.01, ^∗∗∗^*p* < 0.001, ^∗∗∗∗^*p* < 0.0001. Error bars represent means ± SD. Data were analyzed using independent unpaired two-tailed Student's *t*-tests.

## References

[1] Holliday R. (2011). Cohen's pathways of the pulp, 10th edition. *British Dental Journal*.

[2] McLachlan J. L., Sloan A. J., Smith A. J., Landini G., Cooper P. R. (2004). S100 and cytokine expression in caries. *Infection and Immunity*.

[3] Hahn C. L., Liewehr F. R. (2007). Innate immune responses of the dental pulp to caries. *Journal of Endodontia*.

[4] Yonehiro J., Yamashita A., Yoshida Y. (2012). Establishment of an *ex vivo* pulpitis model by co-culturing immortalized dental pulp cells and macrophages. *International Endodontic Journal*.

[5] Suzuki S., Fukuda T., Nagayasu S. (2019). Dental pulp cell-derived powerful inducer of TNF-*α* comprises PKR containing stress granule rich microvesicles. *Scientific Reports*.

[6] Kang R., Tang D. (2012). PKR-dependent inflammatory signals. *Science Signaling*.

[7] Nagendrababu V., Murray P. E., Ordinola-Zapata R. (2021). PRILE 2021 guidelines for reporting laboratory studies in endodontology: explanation and elaboration. *International Endodontic Journal*.

[8] Nagendrababu V., Kishen A., Murray P. E. (2021). PRIASE 2021 guidelines for reporting animal studies in endodontology: explanation and elaboration. *International Endodontic Journal*.

[9] Kamata N., Fujimoto R., Tomonari M., Taki M., Nagayama M., Yasumoto S. (2004). Immortalization of human dental papilla, dental pulp, periodontal ligament cells and gingival fibroblasts by telomerase reverse transcriptase. *Journal of Oral Pathology & Medicine*.

[10] Savitski M. M., Wilhelm M., Hahne H., Kuster B., Bantscheff M. (2015). A scalable approach for protein false discovery rate estimation in large proteomic data sets. *Molecular & Cellular Proteomics*.

[11] Nunes C., Mestre I., Marcelo A., Koppenol R., Matos C. A., Nóbrega C. (2019). MSGP: the first database of the protein components of the mammalian stress granules. *Database*.

[12] Toyoda K., Fukuda T., Sanui T. (2016). Grp78 is critical for amelogenin-induced cell migration in a multipotent clonal human periodontal ligament cell line. *Journal of Cellular Physiology*.

[13] Watanabe Y., Fukuda T., Hayashi C. (2022). Extracellular vesicles derived from GMSCs stimulated with TNF-*α* and IFN-*α* promote M2 macrophage polarization via enhanced CD73 and CD5L expression. *Scientific Reports*.

[14] Fukuda T., Sanui T., Toyoda K. (2013). Identification of novel amelogenin-binding proteins by proteomics analysis. *PLoS One*.

[15] Eba H., Murasawa Y., Iohara K. (2012). The anti-inflammatory effects of matrix metalloproteinase-3 on irreversible pulpitis of mature erupted teeth. *PLoS One*.

[16] Nakao Y., Fukuda T., Zhang Q. (2021). Exosomes from TNF-*α*-treated human gingiva-derived MSCs enhance M2 macrophage polarization and inhibit periodontal bone loss. *Acta Biomaterialia*.

[17] Hayashi C., Fukuda T., Kawakami K. (2022). miR-1260b inhibits periodontal bone loss by targeting ATF6*β* mediated regulation of ER stress. *Frontiers in Cell and Development Biology*.

[18] Patel C. V., Handy I., Goldsmith T., Patel R. C. (2000). PACT, a stress-modulated cellular activator of interferon-induced double-stranded RNA-activated protein kinase, PKR. *The Journal of Biological Chemistry*.

[19] Patel R. C., Sen G. C. (1998). PACT, a protein activator of the interferon-induced protein kinase, PKR. *The EMBO Journal*.

[20] Chukwurah E., Farabaugh K. T., Guan B. J., Ramakrishnan P., Hatzoglou M. (2021). A tale of two proteins: PACT and PKR and their roles in inflammation. *The FEBS Journal*.

[21] Dabo S., Maillard P., Collados Rodriguez M. (2017). Inhibition of the inflammatory response to stress by targeting interaction between PKR and its cellular activator PACT. *Scientific Reports*.

[22] Burnett S. B., Vaughn L. S., Sharma N., Kulkarni R., Patel R. C. (2020). Dystonia 16 (DYT16) mutations in PACT cause dysregulated PKR activation and eIF2*α* signaling leading to a compromised stress response. *Neurobiology of Disease*.

[23] Frederick K., Patel R. C. (2023). Luteolin protects DYT-PRKRA cells from apoptosis by suppressing PKR activation. *Frontiers in Pharmacology*.

[24] Jammi N. V., Whitby L. R., Beal P. A. (2003). Small molecule inhibitors of the RNA-dependent protein kinase. *Biochemical and Biophysical Research Communications*.

[25] Huang J. T., Schneider R. J. (1990). Adenovirus inhibition of cellular protein synthesis is prevented by the drug 2-aminopurine. *Proceedings of the National Academy of Sciences of the United States of America*.

[26] Luo Y., Shang P., Li D. (2017). Luteolin: a flavonoid that has multiple cardio-protective effects and its molecular mechanisms. *Frontiers in Pharmacology*.

[27] Kanda H., Tateya S., Tamori Y. (2006). MCP-1 contributes to macrophage infiltration into adipose tissue, insulin resistance, and hepatic steatosis in obesity. *The Journal of Clinical Investigation*.

[28] Yamamoto H., Ogawa T. (2002). Antimicrobial activity of perilla seed polyphenols against oral pathogenic bacteria. *Bioscience, Biotechnology, and Biochemistry*.

[29] Zhang L., Cai Y., Li L. (2022). Effects of luteolin on biofilm of Trueperella pyogenes and its therapeutic effect on rat endometritis. *International Journal of Molecular Sciences*.

[30] Nabavi S. F., Braidy N., Gortzi O. (2015). Luteolin as an anti-inflammatory and neuroprotective agent: a brief review. *Brain Research Bulletin*.

[31] Simon J. H. S., Glick D. H., Frank A. L. (2013). The relationship of endodontic-periodontic lesions. *Journal of Endodontia*.

