# Supplementary material for: Luteolin Is a Potential Immunomodulating Natural Compound against Pulpal Inflammation
Source: Biomed Res Int. 2024 Jan 25;2024:8864513. doi: 10.1155/2024/8864513 (PMC10834097; doi:10.1155/2024/8864513)
Supplement: Supplementary 1 — Supplementary Table 1: primer sequence used for quantitative RT-PCR. Supplementary Table 2: stress granule-related proteins in MVs from DP-1 detected by proteomic analysis. [file 8864513.f1.docx]

# Supplementary Tables

**Supplementary Table 1.**

Primer sequence used for quantitative RT-PCR

| Gene | Forward Primer | Reverse Primer |
| --- | --- | --- |
| human TNF-α | 5’-CCCAGGGACCTCTCTCTAATCA-3’ | 5’-GCTTGAGGGTTTGCTACAACATG-3’ |
| human MCP-1 | 5’-GACCCCAAGCAGAAGTGGGT-3’ | 5’-GTGTCTGGGGAAAGCTAGGGG-3’ |
| human IL-6 | 5’-ATGAGGAGACTTGCCTGGTG-3’ | 5’-GGTCAGGGGTGGTTATTGCAT-3’ |
| human IL-8 | 5’-ACACTGCGCCAACACAGAAATTA-3’ | 5’-TTTGCTTGAAGTTTCACTGGCATC-3’ |
| human GAPDH | 5’-ATCAAGAAGGTGGTGAAGCAGG-3’ | 5’-GTCATACCAGGAAATGAGC-3’ |
| mouse TNF-α | 5’-GACAGTGACCTGGACTGTGG-3’ | 5’-TGAGACAGAGGCAACCTGAC-3’ |
| mouse 18s rRNA | 5’-GCTTAATTTGACTCAACACGGGA-3’ | 5’-AGCTATCAATCTGTCAATCCTGTC-3’ |

**Supplementary Table 2.**

Stress granule-related proteins in MVs from DP-1 detected by proteomic analysis

| **No.** | **Gene name** | **Protein name** | **Molecular**  **Weight** | **Protein Group Score** | **Score** |
| --- | --- | --- | --- | --- | --- |
| 1 | VCP | Transitional endoplasmic reticulum ATPase | 89 kDa | 0.999750581 | 554656700 |
| 2 | ACTBL2 | Beta-actin-like protein 2 | 42 kDa | 0.9840663 | 525027900 |
| 3 | ATP5F1A | ATP synthase subunit alpha, mitochondrial | 60 kDa | 0.999640715 | 425116200 |
| 4 | ANXA1 | Annexin A1 | 39 kDa | 0.999710116 | 206951400 |
| 5 | ANXA6 | Annexin A6 | 76 kDa | 0.9999245 | 192705000 |
| 6 | LMNA | Prelamin-A/C | 74 kDa | 0.99981669 | 143034300 |
| 7 | HNRNPA2B1 | Heterogeneous nuclear ribonucleoproteins A2/B1 | 37 kDa | 0.999959463 | 122619700 |
| 8 | HSPD1 | 60 kDa heat shock protein, mitochondrial | 61 kDa | 0.999435685 | 121812100 |
| 9 | DSP | Desmoplakin | 332 kDa | 0.999741066 | 113467500 |
| 10 | HSPA9 | Stress-70 protein, mitochondrial | 74 kDa | 0.999570318 | 91301880 |
| 11 | PFN1 | Profilin-1 | 15 kDa | 0.999955399 | 87434690 |
| 12 | CFL1 | Cofilin-1 | 19 kDa | 0.99981387 | 87026590 |
| 13 | DYNC1H1 | Cytoplasmic dynein 1 heavy chain 1 | 532 kDa | 0.999699067 | 79945740 |
| 14 | RPS3 | 40S ribosomal protein S3 | 27 kDa | 0.999475168 | 66972980 |
| 15 | PHB2 | Prohibitin-2 | 33 kDa | 0.999144431 | 61580340 |
| 16 | HNRNPK | Heterogeneous nuclear ribonucleoprotein K | 51 kDa | 0.999672267 | 58992110 |
| 17 | ITGB1 | Integrin beta-1 | 88 kDa | 0.999751553 | 56812400 |
| 18 | RACK1 | Receptor of activated protein C kinase 1 | 35 kDa | 0.999918473 | 55086480 |
| 19 | HSP90AA1 | Heat shock protein HSP 90-alpha | 85 kDa | 0.999894751 | 54535770 |
| 20 | PRDX1 | Peroxiredoxin-1 | 22 kDa | 0.999646194 | 52282360 |
| 21 | TUFM | Elongation factor Tu, mitochondrial | 50 kDa | 0.999811034 | 43633840 |
| 22 | EIF4A1 | Eukaryotic initiation factor 4A-I | 46 kDa | 0.999942643 | 38175880 |
| 23 | TXN | Thioredoxin | 12 kDa | 0.99785082 | 33499860 |
| 24 | SRSF1 | Serine/arginine-rich splicing factor 1 | 28 kDa | 0.99869706 | 25754940 |
| 25 | FLNB | Filamin-B | 278 kDa | 0.999881759 | 23108130 |
| 26 | DCD | Dermcidin | 11 kDa | 0.999115232 | 22777560 |
| 27 | HSPB1 | Heat shock protein beta-1 | 23 kDa | 0.999929821 | 22686890 |
| 28 | UBA1 | Ubiquitin-like modifier-activating enzyme 1 | 118 kDa | 0.999369134 | 21141430 |
| 29 | SRSF3 | Serine/arginine-rich splicing factor 3 | 19 kDa | 0.99873214 | 20963810 |
| 30 | PRDX6 | Peroxiredoxin-6 | 25 kDa | 0.999349399 | 19633940 |
| 31 | TCP1 | T-complex protein 1 subunit alpha | 60 kDa | 0.999837497 | 19567050 |
| 32 | MTHFD1 | C-1-tetrahydrofolate synthase, cytoplasmic | 102 kDa | 0.999491702 | 19523120 |
| 33 | RPS19 | 40S ribosomal protein S19 | 16 kDa | 0.999211597 | 19321430 |
| 34 | CCT3 | T-complex protein 1 subunit gamma | 61 kDa | 0.999219499 | 19167760 |
| 35 | RPS3A | 40S ribosomal protein S3a | 30 kDa | 0.99959412 | 18956710 |
| 36 | STIP1 | Stress-induced-phosphoprotein 1 | 63 kDa | 0.99861757 | 18515940 |
| 37 | DSTN | Destrin | 19 kDa | 0.999302275 | 18216250 |
| 38 | CAP1 | Adenylyl cyclase-associated protein 1 | 52 kDa | 0.999807683 | 17026480 |
| 39 | PCBP2 | Poly(rC)-binding protein 2 | 39 kDa | 0.999716754 | 16714980 |
| 40 | PA2G4 | Proliferation-associated protein 2G4 | 44 kDa | 0.999295259 | 16355220 |
| 41 | CCT6A | T-complex protein 1 subunit zeta | 58 kDa | 0.99856328 | 16278960 |
| 42 | PCNA | Proliferating cell nuclear antigen | 29 kDa | 0.999594415 | 15466060 |
| 43 | CSE1L | Exportin-2 | 110 kDa | 0.999864704 | 14937450 |
| 44 | FBL | rRNA 2'-O-methyltransferase fibrillarin | 34 kDa | 0.999032964 | 14889310 |
| 45 | YWHAB | 14-3-3 protein beta/alpha | 28 kDa | 0.999870672 | 13936130 |
| 46 | DPYSL2 | Dihydropyrimidinase-related protein 2 | 62 kDa | 0.999872983 | 13793040 |
| 47 | DPYSL3 | Dihydropyrimidinase-related protein 3 | 62 kDa | 0.999186953 | 13653540 |
| 48 | SND1 | Staphylococcal nuclease domain-containing protein 1 | 102 kDa | 0.999134923 | 13123630 |
| 49 | PPP2R1A | Serine/threonine-protein phosphatase 2A 65 kDa regulatory subunit A alpha isoform | 65 kDa | 0.999727201 | 12678390 |
| 50 | SFPQ | Splicing factor, proline- and glutamine-rich | 76 kDa | 0.999829484 | 11677140 |
| 51 | PDCD6IP | Programmed cell death 6-interacting protein | 96 kDa | 0.999193373 | 11657940 |
| 52 | RHOA | Transforming protein RhoA | 22 kDa | 0.999240921 | 11466680 |
| 53 | HNRNPA3 | Heterogeneous nuclear ribonucleoprotein A3 | 40 kDa | 0.999514098 | 11216970 |
| 54 | HSPA4 | Heat shock 70 kDa protein 4 | 94 kDa | 0.999094806 | 10413690 |
| 55 | KHDRBS1 | KH domain-containing, RNA-binding, signal transduction-associated protein 1 | 48 kDa | 0.99934285 | 10381020 |
| 56 | PFN2 | Profilin-2 | 15 kDa | 0.999252232 | 10227910 |
| 57 | PKP1 | Plakophilin-1 | 83 kDa | 0.99980268 | 10179360 |
| 58 | EIF3E | Eukaryotic translation initiation factor 3 subunit E | 52 kDa | 0.99799827 | 9967980 |
| 59 | CLIC4 | Chloride intracellular channel protein 4 | 29 kDa | 0.999909861 | 9957916 |
| 60 | GNB2 | Guanine nucleotide-binding protein G(I)/G(S)/G(T) subunit beta-2 | 37 kDa | 0.999480362 | 9556440 |
| 61 | ANXA7 | Annexin A7 | 53 kDa | 0.999242888 | 9466961 |
| 62 | KHSRP | Far upstream element-binding protein 2 | 73 kDa | 0.99817699 | 9009476 |
| 63 | YWHAQ | 14-3-3 protein theta | 28 kDa | 0.999874642 | 8627869 |
| 64 | RNH1 | Ribonuclease inhibitor | 50 kDa | 0.999700155 | 8520999 |
| 65 | ELAVL1 | ELAV-like protein 1 | 36 kDa | 0.99868703 | 8321119 |
| 66 | ACTR1A | Alpha-centractin | 43 kDa | 0.99854145 | 8144186 |
| 67 | CARHSP1 | Calcium-regulated heat-stable protein 1 | 16 kDa | 0.99944345 | 7927793 |
| 68 | ALDH18A1 | Delta-1-pyrroline-5-carboxylate synthase | 87 kDa | 0.999446802 | 7737520 |
| 69 | EIF3L | Eukaryotic translation initiation factor 3 subunit L | 67 kDa | 0.99665641 | 7401877 |
| 70 | PGAM5 | Serine/threonine-protein phosphatase PGAM5, mitochondrial | 32 kDa | 0.99627497 | 7358727 |
| 71 | CDK1 | Cyclin-dependent kinase 1 | 34 kDa | 0.999254294 | 7324815 |
| 72 | USP5 | Ubiquitin carboxyl-terminal hydrolase 5 | 96 kDa | 0.999367664 | 7164722 |
| 73 | UPF1 | Regulator of nonsense transcripts 1 | 124 kDa | 0.999326216 | 7162756 |
| 74 | NUDC | Nuclear migration protein nudC | 38 kDa | 0.99876119 | 7088704 |
| 75 | RTCB | RNA-splicing ligase RtcB homolog | 55 kDa | 0.999828244 | 7048471 |
| 76 | PTGES3 | Prostaglandin E synthase 3 | 19 kDa | 0.999392783 | 6978206 |
| 77 | EIF3A | Eukaryotic translation initiation factor 3 subunit A | 167 kDa | 0.999731133 | 6945544 |
| 78 | KPNB1 | Importin subunit beta-1 | 97 kDa | 0.999752958 | 6907610 |
| 79 | HNRNPD | Heterogeneous nuclear ribonucleoprotein D0 | 38 kDa | 0.999305882 | 6419318 |
| 80 | EIF3B | Eukaryotic translation initiation factor 3 subunit B | 92 kDa | 0.999422144 | 6127972 |
| 81 | KIF5B | Kinesin-1 heavy chain | 110 kDa | 0.999176365 | 5913828 |
| 82 | YARS1 | Tyrosine--tRNA ligase, cytoplasmic | 59 kDa | 0.999177568 | 5906113 |
| 83 | RANBP1 | Ran-specific GTPase-activating protein | 23 kDa | 0.99849784 | 5803080 |
| 84 | TPM1 | Tropomyosin alpha-1 chain | 33 kDa | 0.99807195 | 5718320 |
| 85 | ZC3HAV1 | Zinc finger CCCH-type antiviral protein 1 | 101 kDa | 0.999579937 | 5597701 |
| 86 | MCM7 | DNA replication licensing factor MCM7 | 81 kDa | 0.999264715 | 5572449 |
| 87 | PSMD2 | 26S proteasome non-ATPase regulatory subunit 2 | 100 kDa | 0.999221695 | 5329313 |
| 88 | PDLIM1 | PDZ and LIM domain protein 1 | 36 kDa | 0.999839315 | 5155058 |
| 89 | CSDE1 | Cold shock domain-containing protein E1 | 89 kDa | 0.999097805 | 4905388 |
| 90 | MCM4 | DNA replication licensing factor MCM4 | 97 kDa | 0.99847269 | 4845073 |
| 91 | EWSR1 | RNA-binding protein EWS | 68 kDa | 0.99831635 | 4819479 |
| 92 | EIF3D | Eukaryotic translation initiation factor 3 subunit D | 64 kDa | 0.9989851 | 4666954 |
| 93 | DDX3X | ATP-dependent RNA helicase DDX3X | 73 kDa | 0.999775171 | 4582282 |
| 94 | EIF3H | Eukaryotic translation initiation factor 3 subunit H | 40 kDa | 0.9985854 | 4565168 |
| 95 | EIF4G2 | Eukaryotic translation initiation factor 4 gamma 2 | 102 kDa | 0.99799636 | 4521286 |
| 96 | PRMT1 | Protein arginine N-methyltransferase 1 | 42 kDa | 0.999227726 | 4518810 |
| 97 | NONO | Non-POU domain-containing octamer-binding protein | 54 kDa | 0.999945126 | 4514560 |
| 98 | CNN3 | Calponin-3 | 36 kDa | 0.999608365 | 4504721 |
| 99 | SMC4 | Structural maintenance of chromosomes protein 4 | 147 kDa | 0.99941063 | 4404214 |
| 100 | ARPC1B | Actin-related protein 2/3 complex subunit 1B | 41 kDa | 0.99856332 | 4252189 |
| 101 | MCM5 | DNA replication licensing factor MCM5 | 82 kDa | 0.99869674 | 4224676 |
| 102 | RACGAP1 | Rac GTPase-activating protein 1 | 71 kDa | 0.99636617 | 4176307 |
| 103 | MAP4 | Microtubule-associated protein 4 | 121 kDa | 0.999697943 | 4175844 |
| 104 | MKI67 | Proliferation marker protein Ki-67 | 359 kDa | 0.99899485 | 4175789 |
| 105 | TPM2 | Tropomyosin beta chain | 33 kDa | 0.99807449 | 4172429 |
| 106 | YWHAH | 14-3-3 protein eta | 28 kDa | 0.999108612 | 4163547 |
| 107 | RFC4 | Replication factor C subunit 4 | 40 kDa | 0.99861196 | 3948730 |
| 108 | APEX1 | DNA-(apurinic or apyrimidinic site) lyase | 36 kDa | 0.99888564 | 3886111 |
| 109 | EIF3I | Eukaryotic translation initiation factor 3 subunit I | 37 kDa | 0.999721242 | 3879699 |
| 110 | CIT | Citron Rho-interacting kinase | 231 kDa | 0.999475726 | 3877102 |
| 111 | FSCN1 | Fascin | 55 kDa | 0.99894913 | 3609844 |
| 112 | SRP14 | Signal recognition particle 14 kDa protein | 15 kDa | 0.9981388 | 3570705 |
| 113 | PDLIM4 | PDZ and LIM domain protein 4 | 35 kDa | 0.99879023 | 3501217 |
| 114 | GFPT1 | Glutamine--fructose-6-phosphate aminotransferase [isomerizing] 1 | 79 kDa | 0.999456214 | 3441482 |
| 115 | EIF4H | Eukaryotic translation initiation factor 4H | 27 kDa | 0.99267302 | 3405881 |
| 116 | CTNND1 | Catenin delta-1 | 108 kDa | 0.999565779 | 3399862 |
| 117 | DCTN1 | Dynactin subunit 1 | 142 kDa | 0.999609034 | 3338809 |
| 118 | STAT1 | Signal transducer and activator of transcription 1-alpha/beta | 87 kDa | 0.999155078 | 3317090 |
| 119 | KPNA2 | Importin subunit alpha-1 | 58 kDa | 0.999938185 | 3189651 |
| 120 | STAU1 | Double-stranded RNA-binding protein Staufen homolog 1 | 63 kDa | 0.99708808 | 3101402 |
| 121 | KIF23 | Kinesin-like protein KIF23 | 110 kDa | 0.999790763 | 3091244 |
| 122 | MAGOHB | Protein mago nashi homolog 2 | 17 kDa | 0.9965422 | 3089208 |
| 123 | SNTB2 | Beta-2-syntrophin | 58 kDa | 0.99856727 | 3058570 |
| 124 | ATAD3A | ATPase family AAA domain-containing protein 3A | 71 kDa | 0.99847839 | 3036076 |
| 125 | DDX6 | Probable ATP-dependent RNA helicase DDX6 | 54 kDa | 0.99830823 | 2798453 |
| 126 | NOP58 | Nucleolar protein 58 | 60 kDa | 0.99895811 | 2767014 |
| 127 | CHCHD3 | MICOS complex subunit MIC19 | 26 kDa | 0.999347252 | 2723804 |
| 128 | LARP1 | La-related protein 1 | 124 kDa | 0.99671832 | 2704702 |
| 129 | STRAP | Serine-threonine kinase receptor-associated protein | 38 kDa | 0.99798457 | 2698550 |
| 130 | G3BP1 | Ras GTPase-activating protein-binding protein 1 | 52 kDa | 0.99822312 | 2661214 |
| 131 | FUBP3 | Far upstream element-binding protein 3 | 62 kDa | 0.99704749 | 2630216 |
| 132 | EIF2S2 | Eukaryotic translation initiation factor 2 subunit 2 | 38 kDa | 0.999665686 | 2598635 |
| 133 | YES1 | Tyrosine-protein kinase Yes | 61 kDa | 0.99697124 | 2565681 |
| 134 | PYCR1 | Pyrroline-5-carboxylate reductase 1, mitochondrial | 33 kDa | 0.999765089 | 2507577 |
| 135 | RAB1A | Ras-related protein Rab-1A | 23 kDa | 0.99849868 | 2442288 |
| 136 | PRMT5 | Protein arginine N-methyltransferase 5 | 73 kDa | 0.999793607 | 2441023 |
| 137 | PARP1 | Poly [ADP-ribose] polymerase 1 | 113 kDa | 0.999794008 | 2413854 |
| 138 | TMOD3 | Tropomodulin-3 | 40 kDa | 0.999631717 | 2362193 |
| 139 | DDX1 | ATP-dependent RNA helicase DDX1 | 82 kDa | 0.99668851 | 2359279 |
| 140 | MAPRE1 | Microtubule-associated protein RP/EB family member 1 | 30 kDa | 0.999854064 | 2352657 |
| 141 | RTRAF | RNA transcription, translation and transport factor protein | 28 kDa | 0.99554342 | 2311800 |
| 142 | PALLD | Palladin | 151 kDa | 0.999619455 | 2297907 |
| 143 | ZNF638 | Zinc finger protein 638 | 221 kDa | 0.99624466 | 2286534 |
| 144 | DDX21 | Nucleolar RNA helicase 2 | 87 kDa | 0.99825417 | 2244864 |
| 145 | EIF3J | Eukaryotic translation initiation factor 3 subunit J | 29 kDa | 0.999813588 | 2211090 |
| 146 | S100A9 | Protein S100-A9 | 13 kDa | 0.999926146 | 2143735 |
| 147 | RBBP4 | Histone-binding protein RBBP4 | 48 kDa | 0.999603047 | 2126773 |
| 148 | RSL1D1 | Ribosomal L1 domain-containing protein 1 | 55 kDa | 0.999713737 | 2126313 |
| 149 | SRRT | Serrate RNA effector molecule homolog | 101 kDa | 0.999874328 | 2110921 |
| 150 | EIF3G | Eukaryotic translation initiation factor 3 subunit G | 36 kDa | 0.999904938 | 2090722 |
| 151 | EIF3K | Eukaryotic translation initiation factor 3 subunit K | 25 kDa | 0.99862104 | 2009417 |
| 152 | IPO7 | Importin-7 | 120 kDa | 0.999315698 | 1999900 |
| 153 | HNRNPUL1 | Heterogeneous nuclear ribonucleoprotein U-like protein 1 | 96 kDa | 0.999036574 | 1951401 |
| 154 | MSH6 | DNA mismatch repair protein Msh6 | 153 kDa | 0.999703174 | 1926936 |
| 155 | MARS1 | Methionine--tRNA ligase, cytoplasmic | 101 kDa | 0.999661933 | 1904328 |
| 156 | CAPRIN1 | Caprin-1 | 78 kDa | 0.99798873 | 1830784 |
| 157 | RCC2 | Protein RCC2 | 56 kDa | 0.999201478 | 1823190 |
| 158 | SERBP1 | Plasminogen activator inhibitor 1 RNA-binding protein | 45 kDa | 0.999921169 | 1814736 |
| 159 | PRKRA | Interferon-inducible double-stranded RNA-dependent protein kinase activator A | 34 kDa | 0.99304494 | 1806745 |
| 160 | EIF3F | Eukaryotic translation initiation factor 3 subunit F | 38 kDa | 0.999367127 | 1781072 |
| 161 | EIF2AK2 | Interferon-induced, double-stranded RNA-activated protein kinase | 62 kDa | 0.99567204 | 1778875 |
| 162 | SORBS3 | Vinexin | 75 kDa | 0.99611783 | 1631677 |
| 163 | TUBA4A | Tubulin alpha-4A chain | 50 kDa | 0.9888757 | 1617114 |
| 164 | EIF4G1 | Eukaryotic translation initiation factor 4 gamma 1 | 175 kDa | 0.999070613 | 1549167 |
| 165 | DHX30 | ATP-dependent RNA helicase DHX30 | 134 kDa | 0.99836305 | 1539614 |
| 166 | LSM3 | U6 snRNA-associated Sm-like protein LSm3 | 12 kDa | 0.99560794 | 1536029 |
| 167 | TCEA1 | Transcription elongation factor A protein 1 | 34 kDa | 0.9973969 | 1516079 |
| 168 | KLC1 | Kinesin light chain 1 | 65 kDa | 0.999090131 | 1492438 |
| 169 | NSUN2 | RNA cytosine C(5)-methyltransferase NSUN2 | 86 kDa | 0.999674846 | 1492423 |
| 170 | CDC5L | Cell division cycle 5-like protein | 92 kDa | 0.982277101 | 1474326 |
| 171 | PDLIM5 | PDZ and LIM domain protein 5 | 64 kDa | 0.99824977 | 1413652 |
| 172 | LBR | Delta(14)-sterol reductase LBR | 71 kDa | 0.99898829 | 1347473 |
| 173 | CNOT1 | CCR4-NOT transcription complex subunit 1 | 267 kDa | 0.9875352 | 1310357 |
| 174 | FHL1 | Four and a half LIM domains protein 1 | 36 kDa | 0.99862225 | 1269934 |
| 175 | YBX3 | Y-box-binding protein 3 | 40 kDa | 0.99314691 | 1269254 |
| 176 | IGF2BP1 | Insulin-like growth factor 2 mRNA-binding protein 1 | 63 kDa | 0.99680101 | 1262761 |
| 177 | ETF1 | Eukaryotic peptide chain release factor subunit 1 | 49 kDa | 0.999638998 | 1252460 |
| 178 | CALML5 | Calmodulin-like protein 5 | 16 kDa | 0.99846051 | 1233316 |
| 179 | FAM120A | Constitutive coactivator of PPAR-gamma-like protein 1 | 122 kDa | 0.9961506 | 1217965 |
| 180 | TOMM34 | Mitochondrial import receptor subunit TOM34 | 35 kDa | 0.99898774 | 1207939 |
| 181 | KPNA6 | Importin subunit alpha-7 | 60 kDa | 0.99338721 | 1207601 |
| 182 | MACF1 | Microtubule-actin cross-linking factor 1, isoforms 1/2/3/5 | 838 kDa | 0.9981926 | 1191670 |
| 183 | TNPO1 | Transportin-1 | 102 kDa | 0.999169892 | 1189666 |
| 184 | MYO6 | Unconventional myosin-VI | 150 kDa | 0.99847112 | 1178085 |
| 185 | NUP98 | Nuclear pore complex protein Nup98-Nup96 | 198 kDa | 0.982050899 | 1177785 |
| 186 | CHP1 | Calcineurin B homologous protein 1 | 22 kDa | 0.999116719 | 1133980 |
| 187 | QKI | Protein quaking | 38 kDa | 0.99860951 | 1107065 |
| 188 | HMGB3 | High mobility group protein B3 | 23 kDa | 0.99476823 | 1054190 |
| 189 | IGF2BP3 | Insulin-like growth factor 2 mRNA-binding protein 3 | 64 kDa | 0.999824941 | 1052260 |
| 190 | YARS2 | Tyrosine--tRNA ligase, mitochondrial | 53 kDa | 0.999512748 | 1047862 |
| 191 |  | Mitogen-activated protein kinase kinase kinase kinase 4 | 142 kDa | 0.99675607 | 1043089 |
| 192 | IGF2BP2 | Insulin-like growth factor 2 mRNA-binding protein 2 | 66 kDa | 0.999853668 | 1031493 |
| 193 | DAZAP1 | DAZ-associated protein 1 | 43 kDa | 0.9929213 | 991415.9 |
| 194 | YTHDF3 | YTH domain-containing family protein 3 | 64 kDa | 0.9885123 | 990608.5 |
| 195 | YTHDF1 | YTH domain-containing family protein 1 | 61 kDa | 0.9885123 | 990608.5 |
| 196 | SEC24C | Protein transport protein Sec24C | 118 kDa | 0.999333988 | 985390.7 |
| 197 | BAG3 | BAG family molecular chaperone regulator 3 | 62 kDa | 0.999552125 | 914384.2 |
| 198 | YBX1 | Y-box-binding protein 1 | 36 kDa | 0.99787872 | 913813.1 |
| 199 | CPSF7 | Cleavage and polyadenylation specificity factor subunit 7 | 52 kDa | 0.99888188 | 893678.7 |
| 200 | METAP1 | Methionine aminopeptidase 1 | 43 kDa | 0.99612358 | 886744 |
| 201 | MOV10 | Helicase MOV-10 | 114 kDa | 0.99697038 | 886150.3 |
| 202 | SPATS2L | SPATS2-like protein | 62 kDa | 0.99902287 | 884564.8 |
| 203 | PTBP3 | Polypyrimidine tract-binding protein 3 | 60 kDa | 0.9816845 | 884288.1 |
| 204 | OPTN | Optineurin | 66 kDa | 0.99596152 | 882937.6 |
| 205 | EIF2A | Eukaryotic translation initiation factor 2A | 65 kDa | 0.99891851 | 876766.5 |
| 206 | ROCK1 | Rho-associated protein kinase 1 | 158 kDa | 0.99489327 | 875733.3 |
| 207 | CORO1B | Coronin-1B | 54 kDa | 0.99805359 | 780826.9 |
| 208 | EIF4E | Eukaryotic translation initiation factor 4E | 25 kDa | 0.99731835 | 777059.5 |
| 209 | PRRC2A | Protein PRRC2A | 229 kDa | 0.99706216 | 767145.4 |
| 210 | VASP | Vasodilator-stimulated phosphoprotein | 40 kDa | 0.99707813 | 762304.9 |
| 211 | CDC73 | Parafibromin | 61 kDa | 0.99717032 | 761147.4 |
| 212 | CBFB | Core-binding factor subunit beta | 22 kDa | 0.99243236 | 760606.9 |
| 213 | CAPZA2 | F-actin-capping protein subunit alpha-2 | 33 kDa | 0.999038124 | 760300.5 |
| 214 | MBNL1 | Muscleblind-like protein 1 | 42 kDa | 0.983635999 | 754334.5 |
| 215 | SQSTM1 | Sequestosome-1 | 48 kDa | 0.999825297 | 745675.7 |
| 216 | PPP1R18 | Phostensin | 68 kDa | 0.99877868 | 739784.7 |
| 217 | EDC4 | Enhancer of mRNA-decapping protein 4 | 152 kDa | 0.99666179 | 719730 |
| 218 | U2AF1 | Splicing factor U2AF 35 kDa subunit | 28 kDa | 0.99980413 | 690306.1 |
| 219 | EIF3M | Eukaryotic translation initiation factor 3 subunit M | 43 kDa | 0.999211095 | 670199.1 |
| 220 | SRP9 | Signal recognition particle 9 kDa protein | 10 kDa | 0.999317632 | 667527.4 |
| 221 | SRI | Sorcin | 22 kDa | 0.9948294 | 639942.4 |
| 222 | TNKS1BP1 | 182 kDa tankyrase-1-binding protein | 182 kDa | 0.99266818 | 632150.3 |
| 223 | SMU1 | WD40 repeat-containing protein SMU1 | 58 kDa | 0.99624177 | 631782.6 |
| 224 | ATP2C1 | Calcium-transporting ATPase type 2C member 1 | 101 kDa | 0.99281653 | 628288.5 |
| 225 | DST | Dystonin | 861 kDa | 0.99493543 | 623394.7 |
| 226 | CPSF6 | Cleavage and polyadenylation specificity factor subunit 6 | 59 kDa | 0.99883589 | 617500.8 |
| 227 | RFC3 | Replication factor C subunit 3 | 41 kDa | 0.99603423 | 616383.8 |
| 228 | KANK2 | KN motif and ankyrin repeat domain-containing protein 2 | 91 kDa | 0.99478604 | 611770.7 |
| 229 | PRRC2C | Protein PRRC2C | 317 kDa | 0.99887845 | 608352.6 |
| 230 | SNRPF | Small nuclear ribonucleoprotein F | 10 kDa | 0.99260744 | 586520.7 |
| 231 | HNRNPAB | Heterogeneous nuclear ribonucleoprotein A/B | 36 kDa | 0.99509133 | 566795.7 |
| 232 | DDX47 | Probable ATP-dependent RNA helicase DDX47 | 51 kDa | 0.977701001 | 549300.5 |
| 233 | EIF4B | Eukaryotic translation initiation factor 4B | 69 kDa | 0.999650323 | 530477.6 |
| 234 | DERA | Deoxyribose-phosphate aldolase | 35 kDa | 0.99869606 | 528183.7 |
| 235 | LUZP1 | Leucine zipper protein 1 | 120 kDa | 0.999424223 | 525583.9 |
| 236 | SMARCA1 | Probable global transcription activator SNF2L1 | 123 kDa | 0.9898531 | 520665.1 |
| 237 | NEXN | Nexilin | 81 kDa | 0.9898385 | 484696.2 |
| 238 | FNDC3B | Fibronectin type III domain-containing protein 3B | 133 kDa | 0.9878382 | 483190.9 |
| 239 | CDK2 | Cyclin-dependent kinase 2 | 34 kDa | 0.9982675 | 482887 |
| 240 | FXR1 | Fragile X mental retardation syndrome-related protein 1 | 70 kDa | 0.9877082 | 481771.7 |
| 241 | RCC1 | Regulator of chromosome condensation | 45 kDa | 0.99669067 | 471404.5 |
| 242 | DKC1 | H/ACA ribonucleoprotein complex subunit DKC1 | 58 kDa | 0.999789117 | 466124.4 |
| 243 | POLR2B | DNA-directed RNA polymerase II subunit RPB2 | 134 kDa | 0.99627879 | 443555.4 |
| 244 | NUP205 | Nuclear pore complex protein Nup205 | 228 kDa | 0.99217873 | 434165.9 |
| 245 | RPS6KA3 | Ribosomal protein S6 kinase alpha-3 | 84 kDa | 0.978199299 | 401490.6 |
| 246 | PPME1 | Protein phosphatase methylesterase 1 | 42 kDa | 0.9858177 | 397838.8 |
| 247 | LSM14B | Protein LSM14 homolog B | 42 kDa | 0.99696083 | 366217.7 |
| 248 | CCAR1 | Cell division cycle and apoptosis regulator protein 1 | 133 kDa | 0.99887604 | 363553.9 |
| 249 | DDX19A | ATP-dependent RNA helicase DDX19A | 54 kDa | 0.99335402 | 357643.7 |
| 250 | PURB | Transcriptional activator protein Pur-beta | 33 kDa | 0.9861953 | 349198 |
| 251 | USP10 | Ubiquitin carboxyl-terminal hydrolase 10 | 87 kDa | 0.99455685 | 330373.9 |
| 252 | PAK4 | Serine/threonine-protein kinase PAK 4 | 64 kDa | 0.983382599 | 327490.5 |
| 253 | XRN1 | 5'-3' exoribonuclease 1 | 194 kDa | 0.993338 | 318701.5 |
| 254 | DYNLL2 | Dynein light chain 2, cytoplasmic | 10 kDa | 0.99876228 | 309895.1 |
| 255 | CHORDC1 | Cysteine and histidine-rich domain-containing protein 1 | 37 kDa | 0.9798993 | 306076.7 |
| 256 | SFN | 14-3-3 protein sigma | 28 kDa | 0.99736218 | 304010.9 |
| 257 | IPO8 | Importin-8 | 120 kDa | 0.983165599 | 303214.5 |
| 258 | PELO | Protein pelota homolog | 43 kDa | 0.99861413 | 287752.9 |
| 259 | MTOR | Serine/threonine-protein kinase mTOR | 289 kDa | 0.98496 | 280439.9 |
| 260 | TPT1 | Translationally-controlled tumor protein | 20 kDa | 0.99901848 | 270671 |
| 261 | SRSF4 | Serine/arginine-rich splicing factor 4 | 57 kDa | 0.9872245 | 267471.8 |
| 262 | RAD21 | Double-strand-break repair protein rad21 homolog | 72 kDa | 0.99120846 | 262428.9 |
| 263 | KPNA1 | Importin subunit alpha-5 | 60 kDa | 0.99770386 | 251289.9 |
| 264 | TUBB8 | Tubulin beta-8 chain | 50 kDa | 0.956943899 | 249285.5 |
| 265 | ANP32E | Acidic leucine-rich nuclear phosphoprotein 32 family member E | 31 kDa | 0.9978031 | 243917.1 |
| 266 | NUFIP2 | Nuclear fragile X mental retardation-interacting protein 2 | 76 kDa | 0.99668596 | 242589.5 |
| 267 | DDX58 | Antiviral innate immune response receptor RIG-I | 107 kDa | 0.975356299 | 242084.5 |
| 268 | FAM98A | Protein FAM98A | 55 kDa | 0.99796474 | 219149.4 |
| 269 | LSM14A | Protein LSM14 homolog A | 51 kDa | 0.9564991 | 214989.1 |
| 270 | GSPT1 | Eukaryotic peptide chain release factor GTP-binding subunit ERF3A | 56 kDa | 0.999502024 | 212814.4 |
| 271 | CELF1 | CUGBP Elav-like family member 1 | 52 kDa | 0.99549187 | 203752.8 |
| 272 | PUM1 | Pumilio homolog 1 | 126 kDa | 0.9806174 | 200723.2 |
| 273 | NXF1 | Nuclear RNA export factor 1 | 70 kDa | 0.9841934 | 186626.5 |
| 274 | HNRNPH2 | Heterogeneous nuclear ribonucleoprotein H2 | 49 kDa | 0.999173594 | 174926.7 |
| 275 | SUGP2 | SURP and G-patch domain-containing protein 2 | 120 kDa | 0.99011224 | 171918.4 |
| 276 | IK | Protein Red | 66 kDa | 0.99600631 | 169434.9 |
| 277 | PURA | Transcriptional activator protein Pur-alpha | 35 kDa | 0.973520299 | 168158 |
| 278 | HMGA1 | High mobility group protein HMG-I/HMG-Y | 12 kDa | 0.99692186 | 163127.5 |
| 279 | DNAJC8 | DnaJ homolog subfamily C member 8 | 30 kDa | 0.99058726 | 158161.6 |
| 280 | UBL4A | Ubiquitin-like protein 4A | 18 kDa | 0.955998901 | 155914.4 |
| 281 | DNAJC1 | DnaJ homolog subfamily C member 1 | 64 kDa | 0.99628702 | 155913.6 |
| 282 | MFAP1 | Microfibrillar-associated protein 1 | 52 kDa | 0.99624157 | 153340.2 |
| 283 | ZC3H7A | Zinc finger CCCH domain-containing protein 7A | 111 kDa | 0.99424284 | 136891.2 |
| 284 | TRIP6 | Thyroid receptor-interacting protein 6 | 50 kDa | 0.99502664 | 135020.9 |
| 285 | RGPD3 | RanBP2-like and GRIP domain-containing protein 3 | 197 kDa | 0.99438542 | 119974.6 |
| 286 | DDX50 | ATP-dependent RNA helicase DDX50 | 83 kDa | 0.99577224 | 116544.8 |
| 287 | PDS5B | Sister chromatid cohesion protein PDS5 homolog B | 165 kDa | 0.9809482 | 105171.8 |
| 288 | PARP14 | Protein mono-ADP-ribosyltransferase PARP14 | 203 kDa | 0.9863508 | 100167.9 |
| 289 | AGO2 | Protein argonaute-2 | 97 kDa | 0.99821705 | 92674.13 |
| 290 | PHLDB2 | Pleckstrin homology-like domain family B member 2 | 142 kDa | 0.9793307 | 90739.54 |
| 291 | LEMD3 | Inner nuclear membrane protein Man1 | 100 kDa | 0.9740241 | 85891.36 |
| 292 | TRAF2 | TNF receptor-associated factor 2 | 56 kDa | 0.961729798 | 85141.81 |
| 293 | CSTF1 | Cleavage stimulation factor subunit 1 | 48 kDa | 0.960199501 | 81707.06 |
| 294 | SUN1 | SUN domain-containing protein 1 | 90 kDa | 0.99305356 | 80323.28 |
| 295 | STAU2 | Double-stranded RNA-binding protein Staufen homolog 2 | 63 kDa | 0.99837698 | 78153.79 |
| 296 | PFDN4 | Prefoldin subunit 4 | 15 kDa | 0.99358816 | 76521.81 |
| 297 | G3BP2 | Ras GTPase-activating protein-binding protein 2 | 54 kDa | 0.955081802 | 68676.89 |
| 298 | PUM2 | Pumilio homolog 2 | 114 kDa | 0.99085643 | 68192.99 |
| 299 | UBAP2L | Ubiquitin-associated protein 2-like | 115 kDa | 0.999982415 | 67062.23 |
| 300 | MAPK8 | Mitogen-activated protein kinase 8 | 48 kDa | 0.99006258 | 55571.8 |
| 301 | TUBB3 | Tubulin beta-3 chain | 50 kDa | 0.99456091 | 54264.3 |
| 302 | TAF15 | TATA-binding protein-associated factor 2N | 62 kDa | 0.9874239 | 52979.43 |
| 303 | TUBA1C | Tubulin alpha-1C chain | 50 kDa | 0.984249299 | 48946.28 |
| 304 | AGO1 | Protein argonaute-1 | 97 kDa | 0.99285236 | 45496.51 |
| 305 | CCDC9B | Coiled-coil domain-containing protein 9B | 57 kDa | 0.965011802 | 38968.18 |
| 306 | LARP4 | La-related protein 4 | 81 kDa | 0.9703876 | 32168.02 |
| 307 | SPAG5 | Sperm-associated antigen 5 | 134 kDa | 0.99609925 | 23425.09 |
| 308 | BANF1 | Barrier-to-autointegration factor | 10 kDa | 0.99302723 | 22458.95 |
| 309 | PTK2 | Focal adhesion kinase 1 | 119 kDa | 0.982227501 | 22251.76 |
| 310 | CBX1 | Chromobox protein homolog 1 | 21 kDa | 0.966360699 | 19283.43 |
| 311 | PAWR | PRKC apoptosis WT1 regulator protein | 37 kDa | 0.99629463 | 17765.62 |
| 312 | CTNNA2 | Catenin alpha-2 | 105 kDa | 0.9906063 | 16091.23 |
| 313 | NTMT1 | N-terminal Xaa-Pro-Lys N-methyltransferase 1 | 25 kDa | 0.964695901 | 6564.961 |
