# Supplementary material for: Luteolin Is a Potential Immunomodulating Natural Compound against Pulpal Inflammation
Source: Biomed Res Int. 2024 Jan 25;2024:8864513. doi: 10.1155/2024/8864513 (PMC10834097; doi:10.1155/2024/8864513)
Supplement: Supplementary 2 — Supplementary Figure 1: luteolin downregulates LPS-induced mRNA expressions of MCP-1, IL-6, and IL-8 in dental pulp cells. DP-1 cells were stimulated with E. coli LPS (10 ng/ml) for 3 h with or without pretreatment with luteolin (200 μM) for 24 h (n = 3). ∗∗p < 0.01, ∗∗∗p < 0.001, ∗∗∗∗p < 0.0001. Error bars represent means ± SD. Data were analyzed using independent unpaired two-tailed Student's t-tests. [file 8864513.f2.pdf]

Supplementary Figure 1.

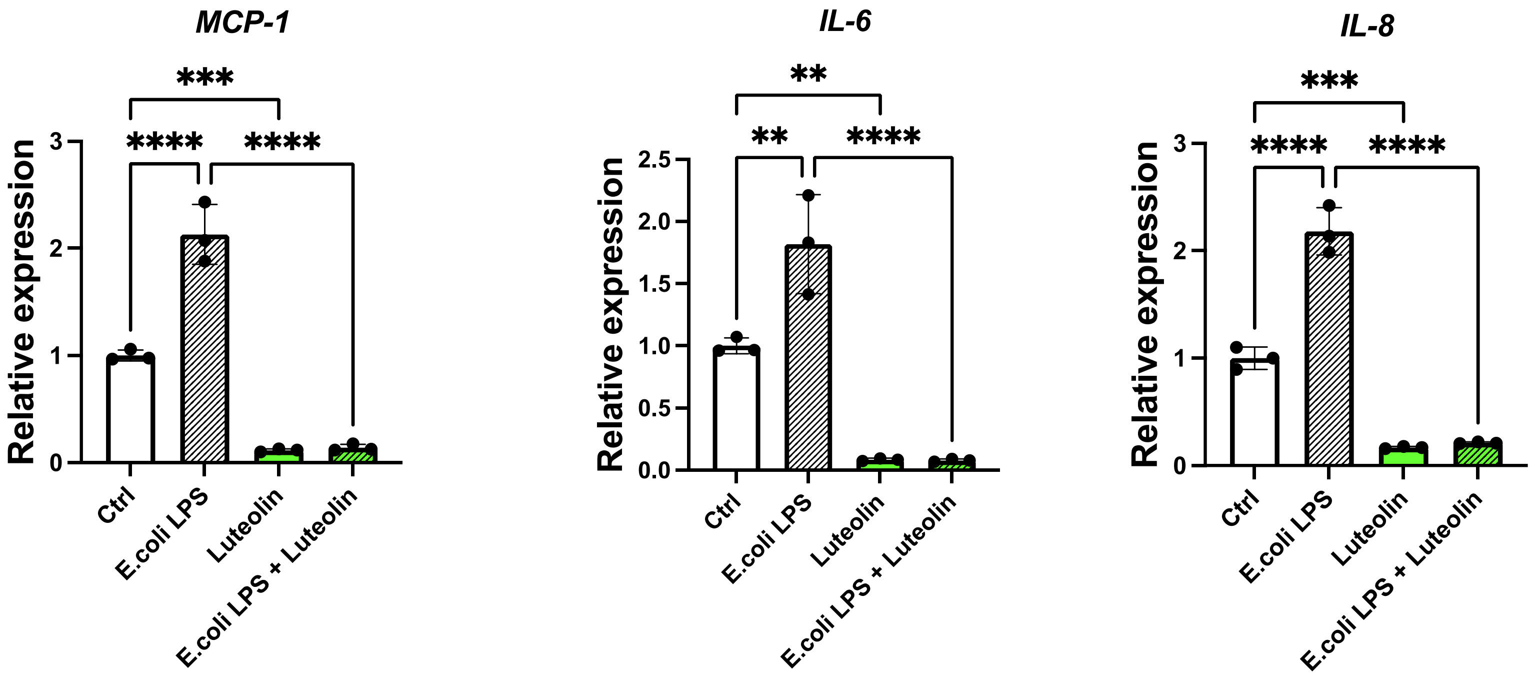

Luteolin downregulates LPS-induced mRNA expressions of MCP-1, IL-6 and IL-8 in dental pulp cells.
DP-1 cells were stimulated with *E. coli* LPS (10 ng/ml) for 3 h with or without pre-treatment with luteolin (200 μM) for 24 h (n = 3).
**p < 0.01, ***p < 0.001, ****p < 0.0001. Error bars represent means ± SD. Data were analyzed using independent unpaired two-tailed Student's t-tests.
